# Endothelial protective factors BMP9 and BMP10 inhibit CCL2 release by human vascular endothelial cells

**DOI:** 10.1242/jcs.239715

**Published:** 2020-07-21

**Authors:** Paul D. Upton, John E. S. Park, Patricia M. De Souza, Rachel J. Davies, Mark J. D. Griffiths, Stephen J. Wort, Nicholas W. Morrell

**Affiliations:** 1 University of Cambridge School of Clinical Medicine, Addenbrooke's/CUHNHSFT and Papworth Hospitals, Cambridge CB2 0QQ, UK; 2 Unit of Critical Care, NHLI, Imperial College, London SW3 6LY, UK

**Keywords:** BMP9, BMP10, CCL2, Endothelial cells, TNF

## Abstract

Bone morphogenetic protein 9 (BMP9) and BMP10 are circulating ligands that mediate endothelial cell (EC) protection via complexes of the type I receptor ALK1 and the type II receptors activin type-IIA receptor (ACTR-IIA) and bone morphogenetic type II receptor (BMPR-II). We previously demonstrated that BMP9 induces the expression of interleukin-6, interleukin-8 and E-selectin in ECs and might influence their interactions with monocytes and neutrophils. We asked whether BMP9 and BMP10 regulate the expression of chemokine (C-C motif) ligand 2 (CCL2), a key chemokine involved in monocyte–macrophage chemoattraction. Here, we show that BMP9 and BMP10 repress basal CCL2 expression and release from human pulmonary artery ECs and aortic ECs. The repression was dependent on ALK1 and co-dependent on ACTR-IIA and BMPR-II. Assessment of canonical Smad signalling indicated a reliance of this response on Smad4. Of note, Smad1/5 signalling contributed only at BMP9 concentrations similar to those in the circulation. In the context of inflammation, BMP9 did not alter the induction of CCL2 by TNF-α. As CCL2 promotes monocyte/macrophage chemotaxis and endothelial permeability, these data support the concept that BMP9 preserves basal endothelial integrity.

## Abstract (precis)

**Summary:** Two proteins found in the blood, BMP9 and BMP10, reduce the production of CCL2, a protein that recruits inflammatory cells to the blood vessel wall.

## INTRODUCTION

Bone morphogenetic protein 9 (BMP9) and BMP10 are circulating members of the transforming growth factor-β (TGF-β) superfamily of ligands that directly activate signalling and mediate functional responses in endothelial cells. BMP9 is the better characterised functionally, having been demonstrated to maintain vascular quiescence (David et al., 2008; Herrera et al., 2014) by reducing vascular permeability (Long et al., 2015), endothelial cell proliferation (David et al., 2008; Scharpfenecker et al., 2007) and protecting against endothelial apoptosis (Long et al., 2015). Although BMP10 is less well characterised with respect to these functions, BMP9 and BMP10 exhibit similar signalling kinetics in endothelial cells (Jiang et al., 2016). Furthermore, BMP9 and BMP10 both circulate in complex with their pro-domains, but unlike most members of the BMP family, their pro-domains do not inhibit activity and could serve to stabilise these proteins in the circulation (Bidart et al., 2012; Jiang et al., 2016).

BMP9 and BMP10 activate endothelial cell signalling via binding to heteromeric complexes of type I and type II receptors. These ligands bind with high affinity to the type I receptor, ALK1 (EC_50_=50 pg/ml), which is expressed at high levels by endothelial cells (Scharpfenecker et al., 2007; David et al., 2007; Upton et al., 2009; Mahmoud et al., 2009). BMP9 signalling also requires the type II receptors, activin type IIA receptor (ACTR-IIA) and bone morphogenetic type II receptor (BMPR-II), which exert differential influences over particular downstream signalling pathways (Upton et al., 2009). BMP10 has broader type II receptor selectivity, also binding to the activin type IIB receptor (ACTR-IIB), although this receptor is not expressed at appreciable levels by endothelial cells (Townson et al., 2012).

Activated BMP receptors signal via C-terminal phosphorylation and activation of the canonical receptor Smad (R-Smad) proteins Smad1, Smad5 and Smad9 (also called Smad8) (Ebisawa et al., 1999; Nohe et al., 2004; Liu et al., 1996; Hoodless et al., 1996), whereas TGF-β receptors typically phosphorylate and activate Smad2 and Smad3. We previously reported that BMP9, signalling via ALK1, stimulates phosphorylation of both Smad1/5 and Smad2 in endothelial cells (Upton et al., 2009). Upon phosphorylation, the R-Smads associate with the common partner Smad, Smad4. The R-Smad:Smad4 complex translocates to the nucleus and associates with other transcription factors to modulate the expression of specific genes (Dennler et al., 1998; Verrecchia et al., 2001; Chen et al., 1997; Hoodless et al., 1999). BMP9 exerts protective effects, such as inhibition of endothelial apoptosis, via Smad1 and Smad5 (Long et al., 2015).

The roles of BMP9 and BMP10 as key regulators of the adult vascular structure are highlighted by the fact that mutations in their endothelial receptors underlie vascular dysplasias. Germ-line mutations in ALK1 underlie hereditary hemorrhagic telangiectasia type 2 (Johnson et al., 1996) and *BMPR2* mutations underlie pulmonary arterial hypertension (PAH) (Lane et al., 2000; Deng et al., 2000; Machado et al., 2006). Although BMP9 and BMP10 serve to promote endothelial integrity under basal conditions, they may also play a role in the effectiveness of the endothelial response to inflammation. We recently reported that BMP9, primarily via ALK1 signalling, increases the lipopolysaccharide-dependent recruitment of neutrophils to pulmonary artery endothelial monolayers under conditions of physiological flow, but without any influence on basal neutrophil recruitment (Appleby et al., 2016). In a similar study from our laboratory examining the impact of BMP9 and BMP10 on tumour necrosis factor-α (TNF-α)-dependent monocyte recruitment to human aortic endothelial cell monolayers, BMP9 enhanced the effect of TNF-α via activation of the ALK2 low affinity receptor (Mitrofan et al., 2017). These studies highlight the possibility that BMP9 and BMP10 exert context-specific influences on endothelial cell responses.

In a previous study, BMP9 induced the expression of inhibitor of DNA-binding-1 (*ID1*) and *ID2*, interleukin-6 (*IL6*), interleukin-8 (*CXCL8*) and E-selectin (*SELE*) by human pulmonary artery endothelial cells (HPAECs). Loss of BMPR-II had little impact on *ID1/2* expression, a result of compensation by ACTR-IIA, whereas loss of BMPR-II almost abolished the induction of interleukin-8 and E-selectin. In contrast, loss of ALK1 globally impacted BMP responses. The induction of *IL6*, *CXCL8* and *SELE* may represent either an inflammatory response profile or a discrete set of regulatory signals mediating vascular function. To address this possibility, we examined the effect of BMP9 on another inflammatory cytokine, chemokine (C-C motif) ligand 2 (CCL2). CCL2 is associated with inflammatory states in several cardiovascular pathologies, primarily via its function as a monocyte/macrophage chemoattractant (Deshmane et al., 2009). CCL2 expression is increased in atherosclerotic plaques (Ylaherttuala et al., 1991; Nelken et al., 1991; Takeya et al., 1993), and circulating CCL2 levels are raised in PAH patients and animal models of PAH (Ikeda et al., 2002; Itoh et al., 2006; Sanchez et al., 2007; Soon et al., 2010). As BMP9 and BMP10 are present in the circulation, we asked whether they mediate the regulation of CCL2 release by endothelial cells under basal states or in the presence of the inflammatory mediator TNF-α.

## RESULTS

### BMP9 and BMP10 inhibit CCL2 production by HPAECs

**Fig. 1.**
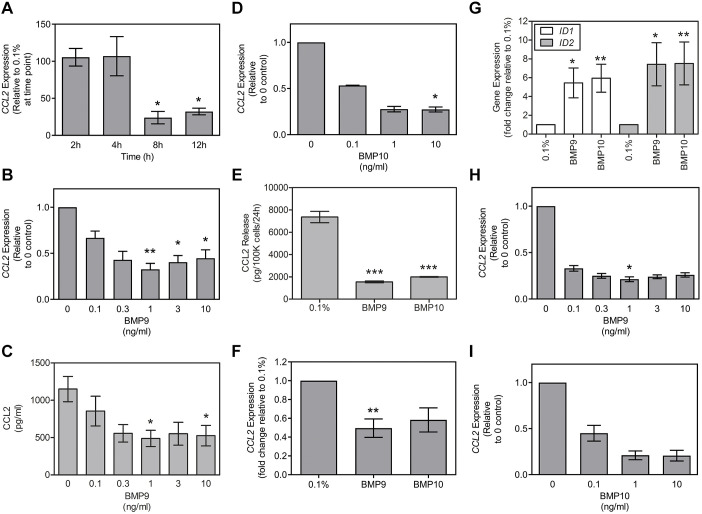
**BMP9 inhibits CCL2 expression and release by endothelial cells.** Confluent HPAECs were serum-restricted for 16 h followed by treatment with BMP9 in 0.1% FBS. (A) HPAECs were treated with 1 ng/ml BMP9 for 2, 4, 8 or 12 h (3 experiments). Data show the fold change relative to 0.1% FBS at each time point. (B) HPAECs were treated with BMP9 (0-10 ng/ml) for 8 h (5 experiments). (C) HPAECs were treated with BMP9 (0-10 ng/ml) for 24 h. CCL2 immunoreactivity of conditioned media was normalized to cell number for each well (*n*=4 wells per treatment) and is representative of 3 experiments. (D) HPAECs were treated with BMP10 (0-10 ng/ml) for 8 h (3 experiments). (E) HPAECs were treated with BMP9 (1 ng/ml) or BMP10 (1 ng/ml) for 24 h. CCL2 immunoreactivity of conditioned media was normalized to cell number for each well. Data (*n*=4 wells per treatment) are representative of 3 experiments. (F,G) HPAECs were treated with BMP9 (1 ng/ml) or BMP10 (1 ng/ml) for 8 h and expression of *CCL2* (F) and *ID1* and *ID2* (G) measured (6 experiments). (H,I) HAECs were treated with BMP9 (0-10 ng/ml) (H) or BMP10 (0-10 ng/ml) (I) for 8 h (3 experiments). All data are expressed as mean±s.e.m. Expression data are normalised to *ACTB* and presented as the fold change relative to control. Significance was calculated using either one-way repeated measures ANOVA with *post-hoc* Tukey's HSD test (B,E-G) or Friedman multiple comparison test with post-hoc Dunn's analysis (C,D,H,I). *P<0.05, **P<0.01, ***P<0.001, compared with control (0.1% FBS without added BMP9 or BMP10).

To establish whether BMP9 altered the expression of CCL2 in HPAECs, cells were treated with control medium (growth medium EBM2 containing 0.1% FBS and antibiotic/antimycotic; henceforth, referred to as 0.1% FBS) alone or supplemented with 1 ng/ml BMP9 for 2, 4, 8 and 12 h. BMP9 significantly reduced the expression of *CCL2* mRNA at 8 and 12 h (Fig. 1A). We then examined the concentration dependence of this response. BMP9 inhibited *CCL2* mRNA expression at 8 h (Fig. 1B) and CCL2 release over a 24 h period (Fig. 1C). BMP9 was effective at concentrations as low as 0.3 ng/ml and maximum inhibition was achieved at 1 ng/ml. Similarly to BMP9, BMP10 also elicited a concentration-dependent inhibition of *CCL2* mRNA expression in HPAECs (Fig. 1D). Both BMP9 and BMP10, at a concentration of 1 ng/ml, repressed CCL2 release (Fig. 1E) and *CCL2* mRNA expression (Fig. 1F), while inducing the expression of the canonical BMP-responsive genes, *ID1* and *ID2* (Fig. 1G). Similarly, both BMP9 (Fig. 1H) and BMP10 (Fig. 1I) inhibited *CCL2* expression in HAECs in a concentration-dependent manner.

### Inhibition of CCL2 is dependent on ALK1 and co-dependent on ACTR-IIA and BMPR-II

**Fig. 2.**
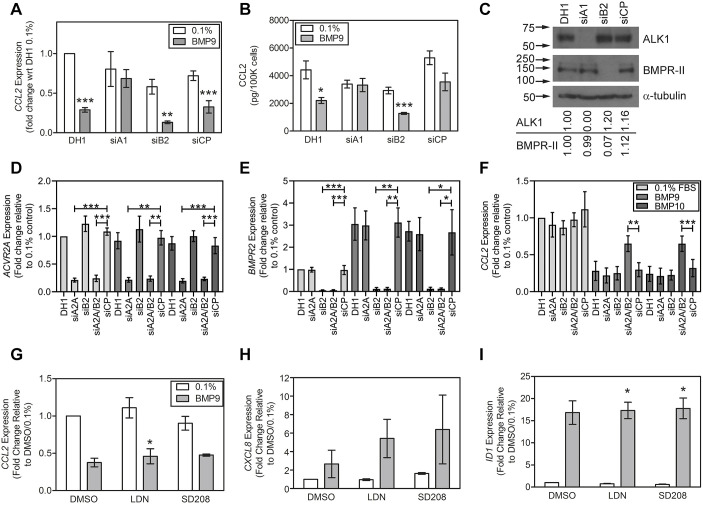
**Reduction of ALK1 attenuates the induction of CCL2 by BMP9, and both ACTR-II and BMPR-II mediate the repression by BMP9 and BMP10.** (A-C) HPAECs were transfected with siRNA for *ALK1* (siA1), *BMPR2* (siB2) or a non-targeting control pool (siCP) using DharmaFECT1 (DH1). (A) HPAECs were treated with 1 ng/ml BMP9 in 0.1% FBS for 8 h. Expression of *CCL2* was normalized to *ACTB*. Data show the fold change relative to DH1/0.1% FBS (4 experiments). (B) HPAECs were treated with 1 ng/ml BMP9 in 0.1% FBS for 24 h. Conditioned media were collected, assayed for CCL2 by ELISA and normalized to cell number for each well. Data (*n*=4 wells per treatment) are from a representative of 4 experiments. (C) Specific reduction of ALK1 and BMPR-II by their respective siRNAs was confirmed by western blotting, the numbers below the blots representing band density ratios relative to α-tubulin normalised to the DH1 control. Arrows indicate the positions of the molecular mass markers (kDa). (D-F) HPAECs were transfected with a non-targeting control siRNA pool (siCP) or siRNAs for *ACVR2A* (siA2A), *BMPR2* (siB2) or both in combination (siA2AB2) using DharmaFECT1 (DH1). HPAECs were treated with 1 ng/ml BMP9 or BMP10 in 0.1% FBS for 8 h. Expression of ACTR-IIA (*ACVR2A*; D), BMPR-II (*BMPR2*; E) and *CCL2* (F) were normalized to *ACTB*. Data show the fold change relative to DH1/0.1% FBS (6 experiments). The key for D-F is provided in F. (G-I) Confluent serum-restricted HPAECs were pretreated with 250 nM LDN-193189 or 2µM SD208 for 1 h followed by 1 ng/ml BMP9 in 0.1% FBS for 8 h for mRNA extraction. Expression of *CCL2* (G), *CXCL8* (H) and *ID1* (I) were determined by qPCR and normalized to *ACTB*. qPCR data are presented as the fold change relative to the DMSO control (1:2500 in 0.1% FBS) (3 experiments). All data are expressed as mean±s.e.m. Significance was calculated using either a paired Students *t*-test (A,B,G-I), comparing with 0.1% FBS control, or one-way repeated measures ANOVA with *post-hoc* Sidak test (D-F), comparing with siCP of same treatment. *P<0.05, **P<0.01, ***P<0.001.

ALK1 and BMPR-II form a BMP9-responsive receptor complex on endothelial cells, so we addressed how loss of ALK1 and BMPR-II, induced through siRNA transfection, might impact on the ability of BMP9 to inhibit CCL2 production by HPAECs. Loss of ALK1 rendered HPAECs resistant to the inhibitory effect of BMP9 on *CCL2* expression (Fig. 2A) and release (Fig. 2B), whereas BMPR-II loss did not. This was also reflected in the *ID1* response (Fig. S1A), consistent with our previous report (Upton et al., 2009). We confirmed specific reduction of ALK1 and BMPR-II proteins (Fig. 2C) and mRNAs (Fig. S1B,C) using their respective siRNAs.

We previously reported that, due to receptor compensation, combined knockdown of ACTR-IIA and BMPR-II is required to attenuate endothelial cell responses to BMP9 (Upton et al., 2009). Therefore, using specific siRNAs for ACTR-IIA (Fig. 2D) and BMPR-II (Fig. 2E), we assessed the relative contributions of ACTR-IIA and BMPR-II to the repression of *CCL2* by BMP9 or BMP10. Knocking down either receptor alone did not alter the repression of CCL2 by BMP9 or BMP10, whereas combined knockdown attenuated this repression (Fig. 2F).

BMP9 can activate low-affinity (>1 ng/ml) ALK2 receptor signalling in endothelial cells (Mitrofan et al., 2017) and ALK1 has previously been reported to couple to ALK5 to mediate TGF-dependent endothelial signalling (Goumans et al., 2002). We therefore assessed the impact of selective inhibition of ALK2/3/6 kinase activities by LDN193189 and ALK5 kinase activity by SD208. Neither inhibitor affected the repression of *CCL2* (Fig. 2G), nor the induction of *CXCL8* (Fig. 2H) or *ID1* (Fig. 2I) by BMP9, confirming that BMP9 did not affect CCL2 via ALK2/3/6 or ALK5.

### BMP9 inhibits CCL2 via Smad4

**Fig. 3.**
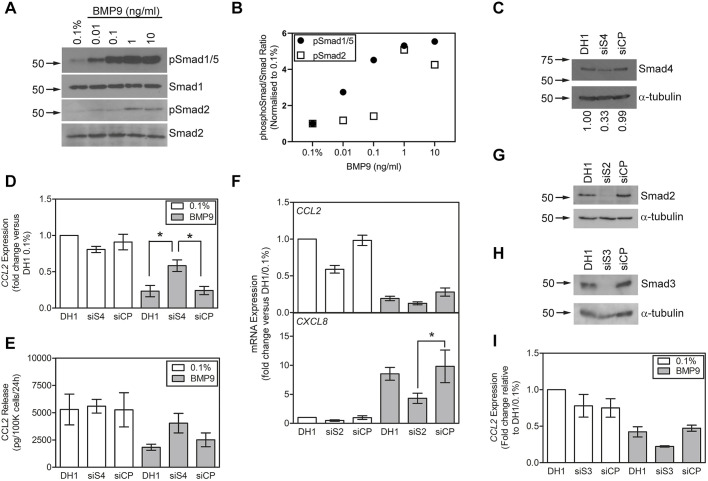
***CCL2* repression by BMP9 in HPAECs is dependent on Smad4, but not Smad2 or Smad3.** (A) Confluent serum-restricted HPAECs were treated with 0.01-10 ng/ml BMP9 in 0.1% FBS for 1 h. Protein lysates were immunoblotted for phospho-Smad1/5, Smad1, phospho-Smad2 or Smad2. Blots are representative of 4 experiments. (B) Quantification of the blots in A, calculated as the ratio of the density of the phospho-Smad band to the Smad band for each sample and normalised to the 0.1% FBS control. (C-E) HPAECs were transfected with *SMAD4* siRNA (siS4) or a non-targeting control pool (siCP) using DharmaFECT1 (DH1). (C) Reduced Smad4 protein was confirmed by western blotting. (D) Confluent serum-restricted HPAECs were treated with 1 ng/ml BMP9 in 0.1% FBS for 8 h (3 experiments). (E) Confluent serum-restricted HPAECs were treated with 1 ng/ml BMP9 in 0.1% FBS for 24 h. CCL2 release was measured by ELISA and normalised to cell number. Data are from a representative of 3 experiments (*n*=4 wells per treatment). (F,G) HPAECs were transfected with *SMAD2* siRNA (siS2) or siCP using DH1 and treated with BMP9 for 8 h as described above. (F) *CCL2* (top panel) and *CXCL8* (bottom panel) expression (3 experiments). (G) Smad2 protein knockdown was confirmed by western blotting. (H,I) HPAECs were transfected with *SMAD3* siRNA (siS3) or siCP using DH1 and treated with BMP9 for 8 h. (H) Smad3 protein knockdown was confirmed by western blotting. (I) *CCL2* expression (3 experiments). For western blots, the migration positions of the relevant protein molecular mass markers (kDa) are indicated by arrows. The numbers below each blot panel represent band density ratios relative to α-tubulin normalised to the DH1 control. All data are expressed as mean±s.e.m. Expression data are normalised to *ACTB* and presented as the fold change relative to DHI/0.1% FBS. Significance was calculated using one-way repeated measures ANOVA with *post-hoc* Tukey’s HSD test. *P<0.05.

We questioned whether BMP9 inhibits CCL2 via Smad-dependent signalling in HPAECs. First, in accordance with our previously reported data (Upton et al., 2009), we demonstrated that BMP9 stimulated C-terminal phosphorylation of Smad1/5 and Smad2 in HPAECs (Fig. 3A). BMP9 stimulated Smad1/5 phosphorylation at concentrations as low as 0.01 ng/ml (Fig. 3A,B). We also observed C-terminal phosphorylation of Smad2 by BMP9 at 1 and 10 ng/ml (Fig. 3A,B).

To determine whether inhibition of CCL2 by BMP9 was Smad-dependent, the effect of *SMAD4* siRNA on BMP9-dependent repression of CCL2 was assessed. The expression of *SMAD4* mRNA was reduced by 88.2±2.3% (Fig. S2A), and reduced Smad4 protein was confirmed by western blotting (Fig. 3C). *SMAD4* siRNA transfection prevented the inhibition of CCL2 expression (Fig. 3D) and release (Fig. 3E) by BMP9 in HPAECs. Furthermore, *SMAD4* knockdown also impaired the induction of *CXCL8* (Fig. S2B) and *ID1* (Fig. S2C) by BMP9 in HPAECs. These data indicate that the repression of CCL2 by BMP9 is via a SMAD4-dependent mechanism.

### CCL2 inhibition by BMP9 is not dependent on Smad2 or Smad3

We previously reported that the induction of *CXCL8* by BMP9 in HPAECs was attenuated by *SMAD2* siRNA in HPAECs (Upton et al., 2009). We therefore sought to determine whether loss of *SMAD2* altered the capacity for BMP9 to inhibit CCL2 expression by HPAECs. HPAECs transfected with *SMAD2* siRNA exhibited a 41±9% (mean±sd) reduction in basal CCL2 expression (Fig. 3F). However, the capacity for BMP9 to block *CCL2* expression was retained with *SMAD2* siRNA, indicating that the BMP9 response is not dependent on Smad2 (Fig. 3F). To ensure that the *SMAD2* siRNA was exerting an effect, we confirmed that *SMAD2* siRNA attenuated *CXCL8* induction by BMP9 in this study (Fig. 3F). Using qPCR, the *SMAD2* mRNA levels were reduced by 97±2% (Fig. S2D) and protein levels were reduced, as assessed by western blotting (Fig. 3G). We considered the possibility that Smad3 mediates the effects of BMP9 via direct signalling or, less likely, as a result of the lack of effect of the ALK5 inhibitor, through an indirect effect on TGF-β signalling. We confirmed that both Smad3 protein (Fig. 3H) and *SMAD3* mRNA (Fig. S2E) were reduced by *SMAD3* siRNA. Unlike *SMAD2* siRNA, *SMAD3* siRNA did not alter basal *CCL2* expression (Fig. 3I). Intriguingly, the inhibition of *CCL2* expression by BMP9 was enhanced by Smad3 loss, implying that Smad3 negatively regulates some BMP9-mediated signalling.

### CCL2 inhibition in HPAECs is dependent on Smad1/5 at BMP9 and BMP10 concentrations that induce high, but not low, affinity signalling

**Fig. 4.**
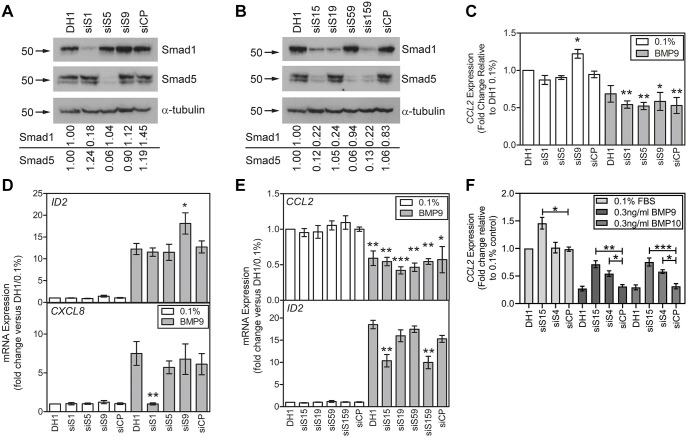
***CCL2* repression by BMP9 in HPAECs is not dependent on Smad1, Smad5 or Smad9.** HPAECs were transfected with siRNAs for *SMAD1* (siS1), *SMAD5* (siS5) or *SMAD9* (siS9) alone or in combination using DharmaFECT1 (DH1). In parallel, cells were transfected with a non-targeting control pool (siCP). (A,B) Knockdown of Smad1 and Smad5 were confirmed by western blotting of cells transfected with siRNAs targeting individual (A) and combinations (B) of Smads. The migration positions of the relevant protein molecular mass markers (kDa) are indicated by arrows. The numbers below each blot panel represent band density ratios relative to α-tubulin normalised to the DH1 control. (C-E) Transfected HPAECs were serum-restricted, followed by treatment with 1 ng/ml BMP9 in 0.1% FBS for 8 h. *CCL2* expression (C) and *ID2* and *CXCL8* expression (D) were determined in cells in which individual Smads were knocked down (5 experiments). *CCL2* and *ID2* expression (E) were determined in HPAECs in which combinations of Smads were knocked down (4 experiments). (F) Transfected HPAECs were serum-restricted, followed by treatment with 0.3 ng/ml BMP9 or BMP10 in 0.1% FBS for 4h (5 experiments). All data are expressed as mean±s.e.m. Expression data are normalised to *ACTB* and presented as the fold change relative to DH1/0.1% FBS. Significance was calculated using one-way repeated measures ANOVA with *post-hoc* Tukey's HSD (C-E) or Sidak (F) test. *P<0.05, **P<0.01, ***P<0.001. For *CCL2*, data were compared with siCP/0.1% FBS. For *ID2* and *CXCL8*, data were compared with siCP/BMP9.

As Smads 1, 5 and 9 are the major receptor Smads associated with BMP signalling, we hypothesised that disruption of BMP-Smad signalling might reverse the repression of *CCL2* by BMP9. We examined the consequences of transfecting individual siRNAs for *SMAD1*, *SMAD5* and *SMAD9*, or combinations of these siRNAs, upon the BMP9 responses in HPAECs. Transfection of individual or combined *SMAD* siRNAs specifically reduced their respective Smad proteins (Fig. 4A,B) and the expression of their mRNAs (Fig. S3A-C). Furthermore, our data demonstrate that BMP9 repressed the expression of *SMAD1* and induced the expression of *SMAD9*, with no significant effect on *SMAD5* (Fig. S3A,B). Transfection of the individual *SMAD* siRNAs did not affect the repression of *CCL2* by BMP9 (Fig. 4C) or the transcriptional induction of *ID1* (Fig. S3C). Intriguingly, we observed that loss of *SMAD9* consistently led to an enhanced *ID2* transcriptional response to BMP9, whereas siRNA for *SMAD1* or *SMAD5* had no effect (Fig. 4D, top panel). Analysis of *CXCL8* induction by BMP9 revealed that Smad1 is essential and necessary for the transcriptional response (Fig. 4D).

As some responses can be co-dependent on Smad1 and Smad5 (Moya et al., 2012), we examined the consequences of combined siRNAs for pairs of *SMAD*s or for all three *SMAD*s. Surprisingly, we did not observe any loss of the repression of *CCL2* expression by BMP9, even when *SMAD1/5/9* were knocked down in combination (Fig. 4E). The Smad1/5 co-dependence of the *ID2* response was highlighted by a reduced response to BMP9 in HPAECs co-transfected with both *SMAD1* and *SMAD5* siRNAs (Fig. 4D), whereas the *ID1* response was not substantially altered (Fig. S3E).

BMP9 and BMP10 induce ALK1 signalling with an EC_50_ of approximately 50 pg/ml (David et al., 2007; Jiang et al., 2016). We questioned whether Smad1/5 might contribute to the high-affinity BMP9 response and whether repression of *CCL2* expression by BMP10 might be Smad dependent. As *CCL2* repression was potent at lower ligand concentrations, we assessed this using 0.3 ng/ml BMP9 or BMP10. Surprisingly, combined knockdown of *SMAD1* and *SMAD5* partially reversed the repression of *CCL2* expression by BMP9 and BMP10 (Fig. 4F). In addition, we showed that BMP10, similarly to BMP9, repressed *CCL2* expression via Smad4 (Fig. 4F). We confirmed the specificity of the knockdowns of these *SMAD*s (Fig. S3F).

### BMP9 and BMP10 do not alter the induction of CCL2 by TNF-α in HPAECs and HAECs

**Fig. 5.**
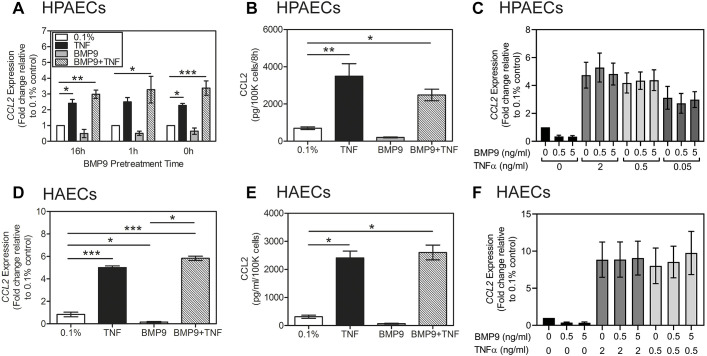
**BMP9 does not affect CCL2 induction by TNF-α in HPAECs and HAECs.** (A) Confluent serum-restricted HPAECs were treated with BMP9 (5 ng/ml) alone or with TNF-α (5 ng/ml) in 0.1% FBS for 6 h. Co-treatments were added without BMP9 pre-incubation or after 1 h or 16 h pre-incubation with 5 ng/ml BMP9 (3 experiments). (B) Confluent serum-restricted HPAECs were treated with BMP9 (5 ng/ml) and TNF-α (5 ng/ml) in 0.1% FBS for 6 h. Conditioned media were assayed for CCL2 by ELISA. Data are from a representative of 3 experiments (*n*=4 wells per treatment). (C) Confluent serum-restricted HPAECs were treated with BMP9 (0-5 ng/ml) and TNF-α (0-2 ng/ml) in 0.1% FBS for 6 h (4 experiments). (D,E) Confluent serum-restricted HAECs were treated with BMP9 (5 ng/ml) alone or with TNF-α (5 ng/ml) in 0.1% FBS for 6 h and assayed for *CCL2* expression (D) (4 experiments) and CCL2 release (E). ELISA data are from a representative of 3 experiments (*n*=4 wells per treatment). (F) Confluent serum-restricted HAECs were treated with BMP9 (0-5 ng/ml) and TNF-α (0-5 ng/ml) in 0.1% FBS for 6 h. All data are expressed as mean±s.e.m. Expression data are normalised to *ACTB* and presented as the fold change relative to 0.1% FBS (no additions). Significance was calculated using Friedman multiple comparisons test with *post-hoc* Dunn’s analysis. *P<0.05, **P<0.01, ***P<0.001, compared with control (0.1% FBS).

Chronic inflammation and overexpression of TNF-α is associated with the pathophysiology of pulmonary arterial hypertension (Hurst et al., 2017) and atherosclerosis (Boesten et al., 2005). As CCL2 production is elevated in inflammation, we examined whether BMP9 could affect the CCL2 response to TNF-α (1 ng/ml). To establish whether BMP9 had a direct effect or might induce a delayed response via transcriptional intermediates, HPAECs were either co-treated with TNF-α and 1 ng/ml BMP9 (0 h) or pretreated with BMP9 for 1 h or 16 h prior to replenishment with TNF-α and 1 ng/ml BMP9. Under all circumstances, the induction of *CCL2* mRNA by TNF-α was not altered by BMP9 whereas basal *CCL2* expression was reduced by BMP9 in all three groups (Fig. 5A). In contrast, the BMP9-dependent induction of *ID1* was repressed by TNF-α (Fig. S4A), whereas the induction of *CXCL8* by BMP9 and TNF-α was additive compared to the response to the individual ligands (Fig. S4B). Analysis of CCL2 release by HPAECs confirmed that BMP9 did not affect CCL2 secretion in response to TNF-α (Fig. 5B). Furthermore, assessment of a range of TNF-α concentrations and a lower BMP9 concentration did not reveal any differential effects on the *CCL2* (Fig. 5C), *ID1* (Fig. S4C) or *CXCL8* (Fig. S4D) responses. We asked whether co-treatment of HAECs with BMP9 and TNF-α would reflect the response observed in HPAECs. Indeed, BMP9 inhibited basal *CCL2* expression (Fig. 5D) and release (Fig. 5E) by HAECs, but did not alter the induction of *CCL2* transcription by TNF-α. Furthermore, TNF-α repressed the BMP9-dependent induction of *ID1* (Fig. S4E) and BMP9 enhanced the induction of *CXCL8* by TNF-α (Fig. S4F), similar to the responses observed in HPAECs. This effect was not dependent on BMP9 or TNF-α concentrations (Fig. 5F; Fig. S4G,H).

## DISCUSSION

BMP9 and BMP10 are circulating ligands that are thought to regulate vascular homeostasis in the adult by promoting endothelial quiescence and monolayer integrity. Here, we report that BMP9 and BMP10 reduce the basal expression and release of the inflammatory cytokine *CCL2* by both HPAECs and HAECs. In contrast, BMP9 and BMP10 do not alter the *CCL2* response to TNF-α and enhance the *CXCL8* response, suggesting that BMP9 might be permissive to the inflammatory response under certain conditions. We also confirm in HPAECs that the basal repression of CCL2 is dependent on ALK1 and Smad4. The Smad2/3 pathways did not contribute, whereas Smad1/5 signalling was involved in the response to BMP9 concentrations reflective of circulating levels. These studies indicate that BMP9 and BMP10 serve to maintain normal endothelial homeostasis by restricting monocyte/macrophage chemoattraction, while being permissive to inflammatory responses.

Studies of BMP9 transcriptional targets in endothelial cells by ourselves and others have reported the induction of secreted factors such as *IL8*, *IL6* and endothelin-1 and cell surface molecules such as E-selectin (Upton et al., 2009; Park et al., 2012; Star et al., 2010). Although these cytokines and E-selectin are implicated in the pathogenesis of chronic inflammation in vascular diseases, the relatively mild induction observed with BMP9 implied that it is not pro-inflammatory at physiological ligand concentrations, confirmed by the relatively weak magnitudes we show in this study of the *CXCL8* responses to BMP9 compared with the response to TNF-α and our previous data regarding E-selectin induction in HAECs (Mitrofan et al., 2017). Here, we analysed the influence of BMP9 and BMP10 on expression of the monocyte chemoattractant protein CCL2 in HPAECs and HAECs. These endothelial cell types are relevant, as CCL2 levels are elevated in PAH patients (Itoh et al., 2006; Soon et al., 2010; Sanchez et al., 2007) and *CCL2* expression is increased in endothelial cells in atherosclerotic plaques (Seino et al., 1995). Our data show that BMP9 and BMP10 potently repress the expression and secretion of CCL2 by both endothelial cell types under basal conditions. This suggests that BMP9 and BMP10 serve to restrict inflammatory cytokine responses in the normal state.

When we assessed the regulation of CCL2 by TNF-α, we did not observe any effect of BMP9 on the response to TNF-α, regardless of the BMP9 or TNF-α concentrations, even though BMP9 consistently repressed basal CCL2 expression and secretion. The interaction between the BMP9 and TNF-α pathways is intriguing, as BMP9 does not influence the induction of *CCL2* by TNF-α, but enhances the *CXCL8* response. We recently reported that BMP9 and BMP10 enhance the TNF-α-dependent recruitment of monocytes to HAEC monolayers under physiological flow (Mitrofan et al., 2017). This response was mediated primarily through the low affinity receptor ALK2, so was only evident at higher BMP9 concentrations (>1.5 ng/ml). In this study, we show that BMP9 represses basal *CCL2* expression at concentrations as low as 0.1 ng/ml. As plasma BMP9 concentrations are reported to be less than 0.5 ng/ml by ELISA (Kienast et al., 2016; Bidart et al., 2012; Nikolic et al., 2019; Hodgson et al., 2020), we suggest that circulating BMP9 and BMP10 serve to reduce monocyte recruitment under normal homeostatic conditions.

In contrast to *CCL2*, the induction of *CXCL8* expression by TNF-α was enhanced by BMP9. Of note, the basal induction of *CXCL8* by BMP9 was weak in comparison to the robust TNF-α response, again suggesting that the BMP9-dependent induction of CXCL8 is unlikely to be inflammatory. CXCL8 can act as a mitogen and chemoattractant for smooth muscle cells, so this could be a mechanism by which BMP9 can stabilise the vessel wall (Yue et al., 1994). This enhancement of the TNF-α response correlates with our recent report showing that BMP9 enhances lipopolysaccharide-dependent neutrophil recruitment to endothelial monolayers under physiological flow, but does not alter basal endothelial adhesion or transmigration (Appleby et al., 2016). Although heightening the TNF-α response appears contradictory to a protective role of BMP9, we suggest this promotes a more effective acute response in the context of tissue damage or infection. Here, we show that TNF-α inhibits BMP9-dependent *ID1/2* signalling and we previously reported that TNF-α represses *BMPR2* in HPAECs (Hurst et al., 2017). Therefore, in the context of pathological chronic inflammation, the continued repression of BMP9 and BMP10 signalling might be detrimental.

BMP9 and BMP10 are high-affinity ligands for the TGF type I receptor ALK1, which is primarily expressed in the vascular endothelium (Scharpfenecker et al., 2007; David et al., 2007; Mahmoud et al., 2009), and loss of ALK1 in HPAECs reduces the Smad phosphorylation and transcriptional responses to BMP9 (Upton et al., 2009). The loss of BMP9 inhibition of *CCL2* we observe with *ALK1* siRNA is consistent with these observations, and the lack of effect of LDN193189 indicates that low-affinity ALK2/3/6 receptors are not involved. We previously demonstrated that the two type II receptors used by BMP9 in HPAECs are BMPR-II and ACTR-IIA (Upton et al., 2009). In contrast to ALK1 insufficiency, loss of BMPR-II reduced the induction of *CXCL8* and *SELE* by BMP9, with minimal impact on Smad1/5 phosphorylation or *ID1/2* gene transcription (Upton et al., 2009). This lack of effect of BMPR-II loss on Smad/ID1/2 signalling is a result of compensation by ACTR-IIA (David et al., 2007; Upton et al., 2009). Here, we show that the repression of *CCL2* by BMP9 or BMP10 is also mediated by both ACTR-IIA and BMPR-II. As BMPR-II loss does not alter the BMP9- or BMP10-dependent inhibition of CCL2 production by HPAECs, increased CCL2 production caused by BMPR-II loss and resistance to BMP9 and BMP10 is unlikely to contribute directly to PAH. However, circulating CCL2 is elevated in PAH patients (Soon et al., 2010; Sanchez et al., 2007; Itoh et al., 2006), so BMPR-II dysfunction, leading to dysregulation of other pathways mediating CCL2 production, may still be involved in the pathology of PAH. For example, pulmonary artery smooth muscle cells from mice expressing a dominant-negative BMPR-II^delEx4^ receptor exhibit increased CCL2 production, implying that CCL2 production from the diseased pulmonary artery media is relevant to the pathogenesis of PAH (Hagen et al., 2007).

The concentration-dependent inhibition of CCL2 by BMP9 seemed to reflect the Smad1/5 C-terminal phosphorylation profile more than the Smad2 phosphorylation. We demonstrate that Smad4 loss renders HPAECs insensitive to CCL2 inhibition in response to BMP9. Previous studies have shown that Smad3-Smad4 mediates TGF-β_1_-mediated CCL2 production (Zhang et al., 1998), so we looked at whether BMP9 interferes with an endogenous TGF-β_1_ or activin pathway, although HPAECs are relatively unresponsive to TGF-β_1_ (Upton et al., 2009). The ALK5 inhibitor SD208 did not affect basal *CCL2* expression or its repression by BMP9. We previously reported that Smad2 is involved in the transcriptional induction of CXCL8 by BMP9 and confirmed the observation in this study (Upton et al., 2009). Smad2 did not mediate the repression of *CCL2* by BMP9, although basal *CCL2* expression appeared to require Smad2. In contrast to Smad2, Smad3 knockdown did not alter basal *CCL2* expression, whereas the inhibitory effect of BMP9 was enhanced with Smad3 knockdown. This implies that Smad3 restricts the inhibition of *CCL2* by BMP9, possibly by enhancing the Smad4-dependent inhibitory response to BMP9.

We propose that Smads 1, 5 or 9, alone or in combination, mediate the repression of *CCL2* via a Smad4-dependent pathway. However, siRNA for either R-Smad alone or in combination did not reverse the inhibition of *CCL2* by 1 ng/ml BMP9. Furthermore, when the impact of Smad1/5 knockdown was assessed in cells treated with 0.3 ng/ml BMP9 or BMP10, the repression of CCL2 was reversed. Again, Smad4 knockout abrogated the inhibitory responses to both BMP9 and BMP10. Therefore, BMP9 and BMP10, at levels measured in human plasma (Nikolic et al., 2019; Hodgson et al., 2020), repress *CCL2* via a Smad1/5- and Smad4-dependent mechanism. However, at a higher concentration of 1 ng/ml BMP9, Smad1/5 knockdown is ineffective with respect to CCL2 repression, whereas Smad4 knockdown still exerts an effect. This reflects our recent data demonstrating that low BMP9 and BMP10 concentrations up to 0.5 ng/ml do not enhance TNF-α-dependent recruitment of monocytes to endothelial monolayers, whereas enhancement is observed with BMP9 and BMP10 concentrations of 1.5 ng/ml and above (Mitrofan et al., 2017). Our observation that combined *SMAD1* and *SMAD5* siRNAs reduced the *ID2* transcriptional response to 1 ng/ml BMP9 demonstrates that the knockdowns were effective. The partial inhibition of the *ID2* response may indicate either sufficient residual Smads to enable a partial response or that a non-Smad pathway contributes to a proportion of the *ID2* response to BMP9. Our data suggest that *ID2* is more reliant upon Smad signals than *ID1*, consistent with a report in epithelial cells indicating that interference with BMP Smad signalling by TGF-β_1_ has a greater effect on *ID2* transcription than on *ID1* (Gronroos et al., 2012). Molecular studies have demonstrated that the promoter binding motifs for Smad4 are in distinctly different DNA regions to the R-Smad sites (Morikawa et al., 2011). Furthermore, a CHIP-seq study in mouse embryonic stem cells showed that only 127 of 2518 Smad4 binding sites were associated with Smad1/5 binding (Fei et al., 2010). At 0.3 ng/ml BMP9, the Smad1/5–Smad4 axis is the key regulatory pathway for CCL2 repression, whereas at 1 ng/ml BMP9, only Smad4 knockdown is effective, suggesting that either a small amount of R-Smad is required for CCL2 repression or the mechanism of the BMP9 response involves Smad4 interacting with non-Smad pathways, such as SMIF/DCP1A (Bai et al., 2002). Intriguingly, BMP9 also regulates the levels of Smad expression, such that BMP9 potently represses the expression of Smad1 and induces Smad9, whereas Smad5 is unaltered. In untreated HPAECs, the expression levels of Smad1, Smad5 and Smad9 are high, moderate and low, respectively, whereas BMP9 treatment equalises the expression levels of the R-Smads. Therefore, loss of BMP9 signalling in endothelial cells may cause an imbalance of Smad expression in favour of Smad1. We also show that BMP9 signalling via Smad1 is essential for the BMP9-dependent induction of *CXCL8*, in addition to a role for Smad2 and our previous report implicating Smad4 (Upton et al., 2009). This implies that BMP9 either induces a Smad1/Smad2/Smad4 complex or that there are separate Smad1 and Smad2 binding sites on the *CXCL8* promoter. These observations highlight a previously unexplored regulation of Smads and Smad-dependent genes by BMP9 in endothelial cells.

BMP9 is considered to be an endothelial protective factor that promotes quiescence in the adult vasculature. The data we present in this study show that BMP9 attenuates CCL2 expression and secretion and support a role for BMP9 in preventing basal monocyte and macrophage recruitment. Furthermore, BMP9 does not impair the endothelial response to inflammation and might prime the endothelium to permit effective responses to infection.

## MATERIALS AND METHODS

### Cell culture

HPAECs (Lonza, Wokingham, UK) were maintained in endothelial growth medium (EGM-2; Lonza) with 2% FBS according to the instructions supplied. Human aortic endothelial cells (HAECs) were purchased from PromoCell (Heidelberg, Germany) and maintained in EGM2-mv (Lonza) with 5% FBS (Invitrogen, Carlsbad, CA). Endothelial cells were cultured at 37°C in a 5% CO_2_ humidified atmosphere and used in experiments at passages 4-6. Cells were confirmed negative for mycoplasma. For studies, endothelial cells were seeded in 24-well or 6-well plates and grown to confluence. Cells were serum-restricted by washing once with 0.1% FBS [EBM2 (Lonza) containing 0.1% FBS and antibiotic/antimycotic (Invitrogen)], followed by incubation in 0.1% FBS. Cells were then treated as described. Cells were regularly tested and confirmed to be mycoplasma-free.

### RNA interference

HPAECs were seeded in 24-well plates (3×10^4^ cells/well) for ELISA studies, 6-well plates (2×10^5^ cells/well) for RNA studies, or 6 cm dishes (4.38×10^5^ cells/dish) for protein extraction and grown for 2 days in EGM-2 (Lonza). Prior to transfection, endothelial cells were incubated in Optimem I (Thermo Fisher) for 3 h. Endothelial cells were transfected with 10 nM siRNA [Dharmacon On-TARGETplus siRNAs for *ALK1* (siA1), *ACVR2A* (siA2A), *BMPR2* (siB2), *SMAD1* (siS1), *SMAD2* (siS2), *SMAD3* (siS3), *SMAD4* (siS4, 20 nM), *SMAD5* (siS5), *SMAD9* (siS9) or siControl Non-targeting Pool (siCP) (Thermo Fisher, Waltham, MA)] in complex with DharmaFECT1 (1 µl/well for 24-well plate, 4 µl/well for 6-well plate or 8.75 µl/dish for 6 cm dish; Thermo Fisher) diluted in Optimem I. Cells were incubated with the complexes for 4 h at 37°C, followed by replacement with EGM-2. For RNA expression studies, cells were incubated with EGM-2 for 28 h followed by serum-restriction in EGM-2 containing 0.1% FBS (0.1% FBS) for 16 h and treated with the relevant ligands (BMP9, BMP10, TNF-α; all from R&D Systems, Abingdon, Oxfordshire, UK) in 0.1% FBS for the times described in the figure legends. Specific reduction of the relevant RNA was quantified using quantitative PCR (qPCR) and, where possible, specific reduction of the relevant protein was also confirmed by western blotting of cells transfected in parallel wells and lysed at the time point when BMP9 was added to the treatment plate.

### Western blotting

Confluent cells in 6 cm dishes were serum-restricted in 0.1% FBS for 16 h. Cells were then treated with recombinant human BMP9 (R&D systems) diluted in 0.1% FBS for 1 h. Cells were snap-frozen and lysed in 150 µl ice-cold radioimmunoassay precipitation buffer [50 mM Tris-HCl pH 8, 150 mM NaCl, 1% (v/v) Igepal CA-630, 0.5% (w/v) sodium deoxycholate, 0.1% (w/v) SDS (all from Sigma-Aldrich, St Louis, MO) containing an EDTA-free protease inhibitor cocktail (Roche Diagnostics, Lewes, East Sussex, UK)]. Lysates were incubated on ice for 30 min and sonicated prior to centrifuging at 12,000×***g*** for 5 min at 4°C. Supernatants were collected and frozen at −20°C until protein assay and western blot analysis. Cell lysates (15-40 µg total protein) were separated on SDS-PAGE gels and proteins transferred to polyvinylidene fluoride membranes by semidry blotting. Blots were then blocked and probed with the relevant antibodies [rabbit monoclonals to C-terminal phospho-Smad1/5, Smad1, C-terminal phospho-Smad2, Smad2 rabbit polyclonal to Smad4 (Cell Signaling Technology, Danvers, MA) or BMPR-II mouse monoclonal (BD Biosciences, Franklin Lakes, NJ)]. The ALK1 rabbit polyclonal was kindly provided by Professor D.A. Marchuk (Duke University, NC). All blots were re-probed with anti-human α-tubulin monoclonal antibody (Sigma-Aldrich). Details of the antibodies used are provided in Table S1. For quantification of blots, the densitometry of the individual bands was analysed using ImageJ. The density for each sample was normalised to the relevant loading control (Smad1 or Smad2 for phospho-Smad signals, α-tubulin for Smad4).

### Quantitative RT-PCR

Confluent HPAECs were quiesced in 0.1% FBS for 16 h followed by treatment with recombinant human BMP9 in 0.1% FBS for 8 h. In some experiments, cells were pretreated with the BMP receptor kinase inhibitor, LDN193189 (250 nM, kindly provided by Dr Paul Yu, Brigham and Women's Hospital, Boston, MA), or the ALK5 inhibitor SD208 (2 µM; Calbiochem) for 1 h prior to the addition of BMP9. DNase-digested total RNA was reverse transcribed using a high capacity cDNA reverse transcription kit (Applied Biosystems). qPCR reactions were amplified on an ABI StepOne Plus (Applied Biosystems) cycler using SYBR Green Jumpstart Taq Readymix (Sigma) with 45ng cDNA and 200nM of each primer. Samples were analysed using Quantitect Primers for *CXCL8*, *SMAD2*, *SMAD3* or *ALK1* (Qiagen) or custom primers for *ACTB* (β-actin), *BMPR2*, *CCL2*, *SMAD1, SMAD4* or *SMAD5* as detailed in Table S2. The efficiency of each primer set was confirmed to be 90-110%. The relative expression of target genes was normalised to β-actin using the ΔΔCT method (Livak and Schmittgen, 2001) and expressed as the fold change relative to the relevant control.

### CCL2 ELISA

HPAECs or HAECs were serum-depleted in 0.1% FBS for 3 h followed by incubation in 0.1% FBS in the presence or absence of exogenous BMP9 or BMP10 as described. After 24 h, supernatants were collected, centrifuged at 1500×***g*** for 10 min at 4°C, aliquoted and stored at −80°C. The cells were trypsinised and counted to allow normalisation of CCL2 values to cell number. Supernatants were assayed using a custom ELISA developed with R&D Duoset reagents (DY279, R&D Systems). High-binding 96-well ELISA plates (Greiner, Kremsmünster, Austria) were coated with mouse monoclonal anti-human CCL2 antibody (0.2 ug/well) in coating buffer (0.15 M sodium carbonate, 0.35 M sodium bicarbonate, pH 9.6) for 2 h at room temperature (RT). Plates were washed with phosphate-buffered saline (PBS; 0.1 M phosphate pH 7.4, 0.137 M NaCl, 2.7 mM KCl) containing 0.05% (v/v) Tween-20 (PBS-T). Plates were then blocked with 5% heat-inactivated FBS (HI-FBS, Thermo Fisher) in PBS-T (5% HI-FBS/PBS-T) for 1 h at RT. Samples (100 µl/well) and recombinant human CCL2 standards (3.9-2000 pg/ml; R&D Systems) were then added and incubated in a humidified chamber overnight at 37°C. Plates were then washed with PBS-T followed by incubation with 45 ng/well biotinylated goat anti-human CCL2 in 5% HI-FBS/PBS-T for 2 h at RT. Plates were washed with PBS-T followed by incubation with ExtrAvidin-alkaline phosphatase (Sigma-Aldrich) diluted 1:400 in 5% HI-FBS/PBS-T for 2 h at RT. Plates were washed with PBS-T followed by water. The ELISA was developed with a colorimetric substrate comprising 1 mg/ml 4-nitrophenyl phosphate disodium salt hexahydrate in 1 M diethanolamine, pH 9.8 containing 0.5 mM MgCl_2_. The assay was developed in the dark at RT and the absorbance measured at 405 nm. Unknown values were extrapolated from the standard curve using a four-parameter curve fit.

### Statistical analysis

Where numbers of experimental repeats were sufficient to confirm normality (*n*=5-6), statistical analysis was performed using unpaired *t*-tests or repeated measures one-way analysis of variance (ANOVA) with post-hoc Tukey's HSD or Sidak analysis to compare individual groups, as stated in the figure legends. For experiments with *n*=3 repeats where normality could not be tested, data were analysed using a Friedman test with Dunn's multiple comparisons.

## Supplementary Material

Supplementary information

Reviewer comments

## References

[JCS239715C1] Appleby S. L., Mitrofan C. G., Crosby A., Hoenderdos K., Lodge K., Upton P. D., Yates C. M., Nash G. B., Chilvers E. R. and Morrell N. W (2016). Bone morphogenetic protein 9 enhances lipopolysaccharide-induced leukocyte recruitment to the vascular endothelium. *J. Immunol.* 197, 3302-3314. 10.4049/jimmunol.1601219 27647829 PMC5104271

[JCS239715C2] Bai R. Y., Koester C., Ouyang T., Hahn S. A., Hammerschmidt M., Peschel C. and Duyster J (2002). SMIF, a Smad4-interacting protein that functions as a co-activator in TGF beta signalling. *Nat. Cell Biol.* 4, 181-190. 10.1038/ncb753 11836524

[JCS239715C3] Bidart M., Ricard N., Levet S., Samson M., Mallet C., David L., Subileau M., Tillet E., Feige J. J. and Bailly S (2012). BMP9 is produced by hepatocytes and circulates mainly in an active mature form complexed to its prodomain. *Cell Mol. Life Sci.* 69, 313-324. 10.1007/s00018-011-0751-1 21710321 PMC11114909

[JCS239715C4] Boesten L. S. M., Zadelaar A. S. M., Van Nieuwkoop A., Gijbels M. J. J., De Winther M. P. J., Havekes L. M. and Van Vlijmen B. J. M (2005). Tumor necrosis factor-alpha promotes atherosclerotic lesion progression in APOE*3-leiden transgenic mice. *Cardiovasc. Res.* 66, 179-185. 10.1016/j.cardiores.2005.01.001 15769461

[JCS239715C5] Chen X., Weisberg E., Fridmacher V., Watanabe M., Naco G. and Whitman M (1997). Smad4 and FAST-1 in the assembly of activin-responsive factor. *Nature* 389, 85-89. 10.1038/38008 9288972

[JCS239715C6] David L., Mallet C., Mazerbourg S., Feige J.-J. and Bailly S (2007). Identification of BMP9 and BMP10 as functional activators of the orphan activin receptor-like kinase 1 (ALK1) in endothelial cells. *Blood* 109, 1953-1961. 10.1182/blood-2006-07-034124 17068149

[JCS239715C7] David L., Mallet C., Keramidas M., Lamandé N., Gasc J.-M., Dupuis-Girod S., Plauchu H., Feige J.-J. and Bailly S (2008). Bone Morphogenetic Protein-9 Is a Circulating Vascular Quiescence Factor. *Circ. Res.* 102, 914-922. 10.1161/CIRCRESAHA.107.165530 18309101 PMC2561062

[JCS239715C8] Deng Z., Morse J. H., Slager S. L., Cuervo N., Moore K. J., Venetos G., Kalachikov S., Cayanis E., Fischer S. G., Barst R. J. et al. (2000). Familial primary pulmonary hypertension (gene PPH1) is caused by mutations in the bone morphogenetic protein receptor-II gene. *Am. J. Hum. Genet.* 67, 737-744. 10.1086/303059 10903931 PMC1287532

[JCS239715C9] Dennler S., Itoh S., Vivien D., Ten Dijke P., Huet S. and Gauthier J. M (1998). Direct binding of Smad3 and Smad4 to critical TGF beta-inducible elements in the promoter of human plasminogen activator inhibitor-type 1 gene. *EMBO J.* 17, 3091-3100. 10.1093/emboj/17.11.3091 9606191 PMC1170648

[JCS239715C10] Deshmane S. L., Kremlev S., Amini S. and Sawaya B. E (2009). Monocyte chemoattractant protein-1 (MCP-1): an overview. *J. Interferon Cytokine Res.* 29, 313-326. 10.1089/jir.2008.0027 19441883 PMC2755091

[JCS239715C11] Ebisawa T., Tada K., Kitajima I., Tojo K., Sampath T. K., Kawabata M., Miyazono K. and Imamura T. (1999). Characterization of bone morphogenetic protein-6 signaling pathways in osteoblast differentiation. *J. Cell Sci.* 112, 3519-3527. 10504300 10.1242/jcs.112.20.3519

[JCS239715C12] Fei T., Xia K., Li Z. W., Zhou B., Zhu S. S., Chen H., Zhang J. P., Chen Z., Xiao H. S., Han J.-D. J. and et al. (2010). Genome-wide mapping of SMAD target genes reveals the role of BMP signaling in embryonic stem cell fate determination. *Genome Res.* 20, 36-44. 10.1101/gr.092114.109 19926752 PMC2798829

[JCS239715C13] Goumans M.-J., Valdimarsdottir G., Itoh S., Rosendahl A., Sideras P. and Ten Dijke P (2002). Balancing the activation state of the endothelium via two distinct TGF-beta type I receptors. *EMBO J.* 21, 1743-1753. 10.1093/emboj/21.7.1743 11927558 PMC125949

[JCS239715C14] Gronroos E., Kingston I. J., Ramachandran A., Randall R. A., Vizan P. and Hill C. S (2012). Transforming growth factor beta inhibits bone morphogenetic protein-induced transcription through novel phosphorylated Smad1/5-Smad3 complexes. *Mol. Cell Biol.* 32, 2904-2916. 10.1128/MCB.00231-12 22615489 PMC3416179

[JCS239715C15] Hagen M., Fagan K., Steudel W., Carr M., Lane K., Rodman D. M. and West J (2007). Interaction of interleukin-6 and the BMP pathway in pulmonary smooth muscle. *Am. J. Physiol Lung Cell Mol. Physiol.* 292, L1473-L1479. 10.1152/ajplung.00197.2006 17322283

[JCS239715C16] Herrera B., Dooley S. and Breitkopf-Heinlein K (2014). Potential roles of bone morphogenetic protein (BMP)-9 in human liver diseases. *Int. J. Mol. Sci.* 15, 5199-5220. 10.3390/ijms15045199 24670474 PMC4013558

[JCS239715C17] Hodgson J., Swietlik E. M., Salmon R. M., Hadinnapola C., Nikolic I., Wharton J., Guo J., Liley J., Haimel M., Bleda M. et al. (2020). Characterization of GDF2 mutations and levels of BMP9 and BMP10 in pulmonary arterial hypertension. *Am. J. Respir. Crit. Care. Med.* 201, 575-585. 10.1164/rccm.201906-1141OC 31661308 PMC7047445

[JCS239715C18] Hoodless P. A., Haerry T., Abdollah S., Stapleton M., O'connor M. B., Attisano L. and Wrana J. L (1996). MADR1, a MAD-related protein that functions in BMP2 signaling pathways. *Cell* 85, 489-500. 10.1016/S0092-8674(00)81250-7 8653785

[JCS239715C19] Hoodless P. A., Tsukazaki T., Nishimatsu S., Attisano L., Wrana J. L. and Thomsen G. H (1999). Dominant-negative Smad2 mutants inhibit activin/Vg1 signaling and disrupt axis formation in Xenopus. *Dev. Biol.* 207, 364-379. 10.1006/dbio.1998.9168 10068469

[JCS239715C20] Hurst L. A., Dunmore B. J., Long L., Crosby A., Al-Lamki R., Deighton J., Southwood M., Yang X. D., Nikolic M. Z., Herrera B. et al. (2017). TNFα drives pulmonary arterial hypertension by suppressing the BMP type-II receptor and altering NOTCH signalling. *Nat. Commun.* 8, 14079 10.1038/ncomms14079 28084316 PMC5241886

[JCS239715C21] Ikeda Y., Yonemitsu Y., Kataoka C., Kitamoto S., Yamaoka T., Nishida K., Takeshita A., Egashira K. and Sueishi K (2002). Anti-monocyte chemoattractant protein-1 gene therapy attenuates pulmonary hypertension in rats. *Am. J. Physiol. Heart Circ. Physiol.* 283, H2021-H2028. 10.1152/ajpheart.00919.2001 12384481

[JCS239715C22] Itoh T., Nagaya N., Ishibashi-Ueda H., Kyotani S., Oya H., Sakamaki F., Kimura H. and Nakanishi N (2006). Increased plasma monocyte chemoattractant protein-1 level in idiopathic pulmonary arterial hypertension. *Respirology* 11, 158-163. 10.1111/j.1440-1843.2006.00821.x 16548900

[JCS239715C23] Jiang H., Salmon R. M., Upton P. D., Wei Z., Lawera A., Davenport A. P., Morrell N. W. and Li W (2016). The prodomain-bound form of bone morphogenetic protein 10 is biologically active on endothelial cells. *J. Biol. Chem.* 291, 2954-2966. 10.1074/jbc.M115.683292 26631724 PMC4742757

[JCS239715C24] Johnson D. W., Berg J. N., Baldwin M. A., Gallione C. J., Marondel I., Yoon S. J., Stenzel T. T., Speer M., Pericak-Vance M. A., Diamond A. et al. (1996). Mutations in the activin receptor-like kinase 1 gene in hereditary haemorrhagic telangiectasia type 2. *Nat. Genet.* 13, 189-195. 10.1038/ng0696-189 8640225

[JCS239715C25] Kienast Y., Jucknischke U., Scheiblich S., Thier M., De Wouters M., Haas A., Lehmann C., Brand V., Bernicke D., Honold K. and et al. (2016). Rapid activation of bone morphogenic protein 9 by receptor-mediated displacement of pro-domains. *J. Biol. Chem.* 291, 3395-3410. 10.1074/jbc.M115.680009 26677222 PMC4751383

[JCS239715C26] Lane K. B., Machado R. D., Pauciulo M. W., Thomson J. R., Phillips J. A., Loyd J. E., Nichols W. C. and Trembath R. C. (2000). Heterozygous germline mutations in BMPR2, encoding a TGF-beta receptor, cause familial primary pulmonary hypertension. *Nat. Genet.* 26, 81-84. 10.1038/79226 10973254

[JCS239715C27] Liu F., Hata A., Baker J. C., Doody J., Cárcamo J., Harland R. M. and Massagué J (1996). A human Mad protein acting as a BMP-regulated transcriptional activator. *Nature* 381, 620-623. 10.1038/381620a0 8637600

[JCS239715C28] Livak K. J. and Schmittgen T. D (2001). Analysis of relative gene expression data using real-time quantitative PCR and the 2−ΔΔCT Method. *Methods* 25, 402-408. 10.1006/meth.2001.1262 11846609

[JCS239715C29] Long L., Ormiston M. L., Yang X., Southwood M., Gräf S., Machado R. D., Mueller M., Kinzel B., Yung L. M., Wilkinson J. M. et al. (2015). Selective enhancement of endothelial BMPR-II with BMP9 reverses pulmonary arterial hypertension. *Nat. Med.* 21, 777-785. 10.1038/nm.3877 26076038 PMC4496295

[JCS239715C30] Machado R. D., Aldred M. A., James V., Harrison R. E., Patel B., Schwalbe E. C., Gruenig E., Janssen B., Koehler R., Seeger W. et al. (2006). Mutations of the TGF-beta type II receptor BMPR2 in pulmonary arterial hypertension. *Hum. Mutat.* 27, 121-132. 10.1002/humu.20285 16429395

[JCS239715C31] Mahmoud M., Borthwick G. M., Hislop A. A. and Arthur H. M (2009). Endoglin and activin receptor-like-kinase 1 are co-expressed in the distal vessels of the lung: implications for two familial vascular dysplasias, HHT and PAH. *Lab. Invest.* 89, 15-25. 10.1038/labinvest.2008.112 19015642

[JCS239715C32] Mitrofan C.-G., Appleby S. L., Nash G. B., Mallat Z., Chilvers E. R., Upton P. D. and Morrell N. W (2017). Bone morphogenetic protein 9 (BMP9) and BMP10 enhance tumor necrosis factor-alpha-induced monocyte recruitment to the vascular endothelium mainly via activin receptor-like kinase 2. *J. Biol. Chem.* 292, 13714-13726. 10.1074/jbc.M117.778506 28646109 PMC5566526

[JCS239715C33] Morikawa M., Koinuma D., Tsutsumi S., Vasilaki E., Kanki Y., Heldin C. H., Aburatani H. and Miyazono K (2011). ChIP-seq reveals cell type-specific binding patterns of BMP-specific Smads and a novel binding motif. *Nucleic Acids Res.* 39, 8712-8727. 10.1093/nar/gkr572 21764776 PMC3203580

[JCS239715C34] Moya I. M., Umans L., Maas E., Pereira P. N. G., Beets K., Francis A., Sents W., Robertson E. J., Mummery C. L., Huylebroeck D. and et al. (2012). Stalk cell phenotype depends on integration of Notch and Smad1/5 signaling cascades. *Dev. Cell* 22, 501-514. 10.1016/j.devcel.2012.01.007 22364862 PMC4544746

[JCS239715C35] Nelken N. A., Coughlin S. R., Gordon D. and Wilcox J. N (1991). Monocyte chemoattractant protein-1 in human atheromatous plaques. *J. Clin. Investig.* 88, 1121-1127. 10.1172/JCI115411 1843454 PMC295565

[JCS239715C36] Nikolic I., Yung L.-M., Yang P., Malhotra R., Paskin-Flerlage S. D., Dinter T., Bocobo G. A., Tumelty K. E., Faugno A. J., Troncone L. et al. (2019). Bone morphogenetic protein 9 is a mechanistic biomarker of portopulmonary hypertension. *Am. J. Respir. Crit. Care Med.* 199, 891-902. 10.1164/rccm.201807-1236OC 30312106 PMC6444661

[JCS239715C37] Nohe A., Keating E., Knaus P. and Petersen N. O (2004). Signal transduction of bone morphogenetic protein receptors. *Cell. Signal.* 16, 291-299. 10.1016/j.cellsig.2003.08.011 14687659

[JCS239715C38] Park J. E., Shao D., Upton P. D., Desouza P., Adcock I. M., Davies R. J., Morrell N. W., Griffiths M. J. and Wort S. J (2012). BMP-9 induced endothelial cell tubule formation and inhibition of migration involves Smad1 driven endothelin-1 production. *PLoS One* 7, e30075 10.1371/journal.pone.0030075 22299030 PMC3267722

[JCS239715C39] Sanchez O., Marcos E., Perros F., Fadel E., Tu L., Humbert M., Dartevelle P., Gerald S., Adnot S. and Eddahibi S (2007). Role of endothelium-derived CC chemokine ligand 2 in idiopathic pulmonary arterial hypertension. *Am. J. Respir. Crit. Care. Med.* 176, 1041-1047. 10.1164/rccm.200610-1559OC 17823354

[JCS239715C40] Scharpfenecker M., Van D. M., Liu Z., Van Bezooijen R. L., Zhao Q., Pukac L., Lowik C. W. and ten Dijke P. (2007). BMP-9 signals via ALK1 and inhibits bFGF-induced endothelial cell proliferation and VEGF-stimulated angiogenesis. *J. Cell Sci.* 120, 964-972. 10.1242/jcs.002949 17311849

[JCS239715C41] Seino Y., Ikeda U., Takahashi M., Hojo Y., Irokawa M., Kasahara T. and Shimada K (1995). Expression of monocyte chemoattractant Protein-1 in vascular tissue. *Cytokine* 7, 575-579. 10.1006/cyto.1995.0078 8580375

[JCS239715C42] Soon E., Holmes A. M., Treacy C. M., Doughty N. J., Southgate L., Machado R. D., Trembath R. C., Jennings S., Barker L., Nicklin P. et al. (2010). Elevated levels of inflammatory cytokines predict survival in idiopathic and familial pulmonary arterial hypertension. *Circulation* 122, 920-927. 10.1161/CIRCULATIONAHA.109.933762 20713898

[JCS239715C43] Star G. P., Giovinazzo M. and Langleben D (2010). Bone morphogenic protein-9 stimulates endothelin-1 release from human pulmonary microvascular endothelial cells: A potential mechanism for elevated ET-1 levels in pulmonary arterial hypertension. *Microvasc. Res.* 80, 349-354. 10.1016/j.mvr.2010.05.010 20594999

[JCS239715C44] Takeya M., Yoshimura T., Leonard E. J. and Takahashi K (1993). Detection of Monocyte Chemoattractant Protein-1 in Human Atherosclerotic Lesions by an Anti-Monocyte Chemoattractant Protein-1 Monoclonal-Antibody. *Hum. Pathol.* 24, 534-539. 10.1016/0046-8177(93)90166-E 7684023

[JCS239715C45] Townson S. A., Martinez-Hackert E., Greppi C., Lowden P., Sako D., Liu J., Ucran J. A., Liharska K., Underwood K. W., Seehra J. et al. (2012). Specificity and structure of a high affinity activin receptor-like kinase 1 (ALK1) signaling complex. *J. Biol. Chem.* 287, 27313-27325. 10.1074/jbc.M112.377960 22718755 PMC3431715

[JCS239715C46] Upton P. D., Davies R. J., Trembath R. C. and Morrell N. W (2009). Bone morphogenetic protein (BMP) and activin type II receptors balance BMP9 signals mediated by activin receptor-like kinase-1 in human pulmonary artery endothelial cells. *J. Biol. Chem.* 284, 15794-15804. 10.1074/jbc.M109.002881 19366699 PMC2708876

[JCS239715C47] Verrecchia F., Vindevoghel L., Lechleider R. J., Uitto J., Roberts A. B. and Mauviel A (2001). Smad3/AP-1 interactions control transcriptional responses to TGF-beta in a promoter-specific manner. *Oncogene* 20, 3332-3340. 10.1038/sj.onc.1204448 11423983

[JCS239715C48] Ylaherttuala S., Lipton B. A., Rosenfeld M. E., Sarkioja T., Yoshimura T., Leonard E. J., Witztum J. L. and Steinberg D. (1991). Expression of monocyte chemoattractant protein-1 in macrophage-rich areas of human and rabbit atherosclerotic lesions. *Proc. Natl. Acad. Sci. U.S.A.* 88, 5252-5256. 10.1073/pnas.88.12.5252 2052604 PMC51850

[JCS239715C49] Yue T. L., Wang X., Sung C. P., Olson B., Mckenna P. J., Gu J. L. and Feuerstein G. Z (1994). Interleukin-8. A mitogen and chemoattractant for vascular smooth muscle cells. *Circ. Res.* 75, 1-7. 10.1161/01.RES.75.1.1 8013067

[JCS239715C50] Zhang Y., Feng X.-H. and Derynck R (1998). Smad3 and Smad4 cooperate with c-Jun/c-Fos to mediate TGF-beta-induced transcription. *Nature* 394, 909-913. 10.1038/29814 9732876

