# Supplementary material for: Endothelial protective factors BMP9 and BMP10 inhibit CCL2 release by human vascular endothelial cells
Source: J Cell Sci. 2020 Jul 21;133(14):jcs239715. doi: 10.1242/jcs.239715 (PMC7390625; doi:10.1242/jcs.239715)
Supplement: Supplementary information [file joces-133-239715-s1.pdf]

DATA SUPPLEMENT

Table S1: Details of antibodies used in this study

Target	Cat No/Clone	Dilution	Source
ALK1	Rabbit 11313	1:750	Prof. D. Marchuk
BMPR-II	BD612292	1:400	Cell signalling
phospho-Smad1/5	#9516	1:1000	Cell signalling
Smad1	#9512	1:1000	Cell signalling
phospho-Smad2	#3108	1:500	Cell signalling
Smad2	#3122	1:750	Cell signalling
Smad3	#9523	1:750	Cell signalling
Smad4	#9515	1:1000	Cell signalling
Smad5	ab40771	1:1000	Abcam
α-tubulin	T6199 (Clone DM1A)	1:5000	Sigma

Table S2: Sequences of QPCR Primers and Quantitect™ Catalogue Numbers

Target (Protein)	Primer sequences/Quantitect™ (QT) Catalogue Numbers
ACTB	For: 5’-GCACCACACCTTCTACAATGA-3’<br>Rev: 5’-GTCATCTTCTCGCGGTTGGC-3’
BMPR2	For: 5’-CAAATCTGTGAGCCCAACAGTCAA-3’<br>Rev: 5’-GAGGAAGAATAATCTGGATAAGGACCAAT-3’
CCL2	For: 5’-TCAGCCAGATGCAATCAATGCC-3’<br>Rev: 5’-GCGAGCCTCTGCACTGAGATCT=3’
SMAD1	For: 5’-TAGAAAGCCCTGTACTTCCTC-3’<br>Rev: 5’-GGTTGCTGGAAAGAATCTGG-3’
SMAD4	For: 5’-GACATTACTGGCCTGTTCAC-3’<br>Rev: 5′-GCAATGGAACACCAATACTCAG-3’
SMAD5	For: 5’-GAGAGTCCAGTCTTACCTCC-3’<br>Rev: 5’-GGAAAGAATCTGGAAACGTG-3’
SMAD9	For: 5’-TACTGTCGCGTGTGGCGCTG-3’<br>Rev: 5’-AGCACAGGAGGCAGTACTGGAG-3’
ACVR2A (ACTR-IIA)	QT00077749
ACVRL1 (ALK1)	QT00050351
SMAD2	QT00004207
SMAD3	QT00008729

Journal of Cell Science • Supplementary information

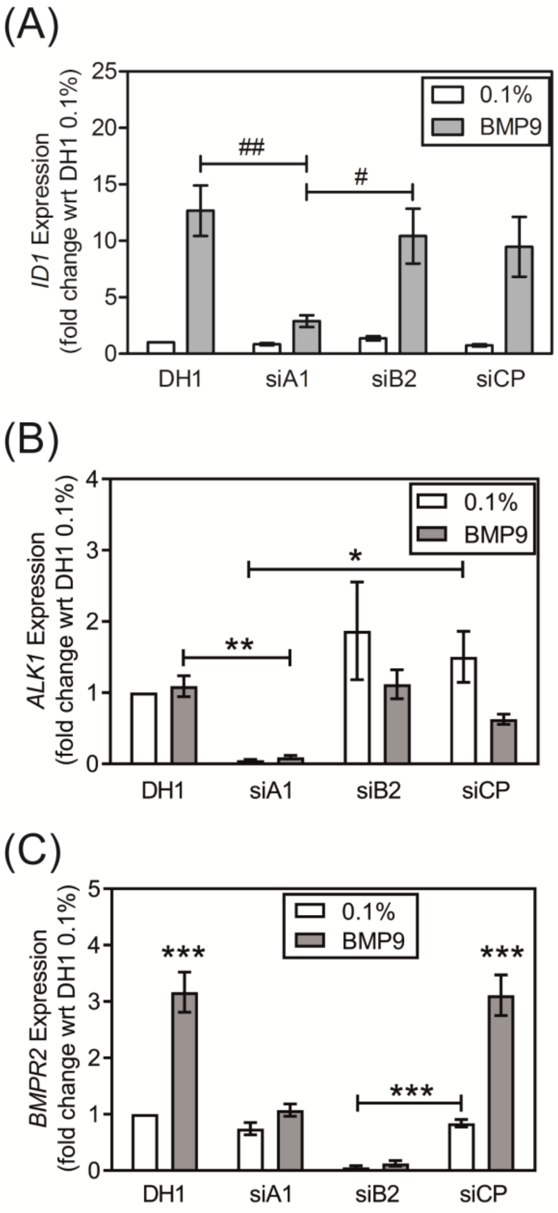

Figure S1. siRNA for *ALK1* and *BMPR2*.

(A-C) HPAECs were transfected with siRNA for *ALK1* (siA1), *BMPR2* (siB2) or a non-targeting control pool (siCP) using DharmaFECT1™ (DH1) followed by treatment with 1ng/ml BMP9 in 0.1% FBS (0.1%) for 8h. The expression of (A) *ID1*, (B) *ALK1* and (C) *BMPR2* were normalized to *ACTB* and are expressed as the fold change relative to DH1/0.1%. Data are mean ± SEM of 5 experiments. ## P<0.01, #P<0.05, one-way ANOVA. *P<0.05, **P<0.01 and ***P<0.001 compared to 0.1% FBS control, students t-test.

Journal of Cell Science • Supplementary information

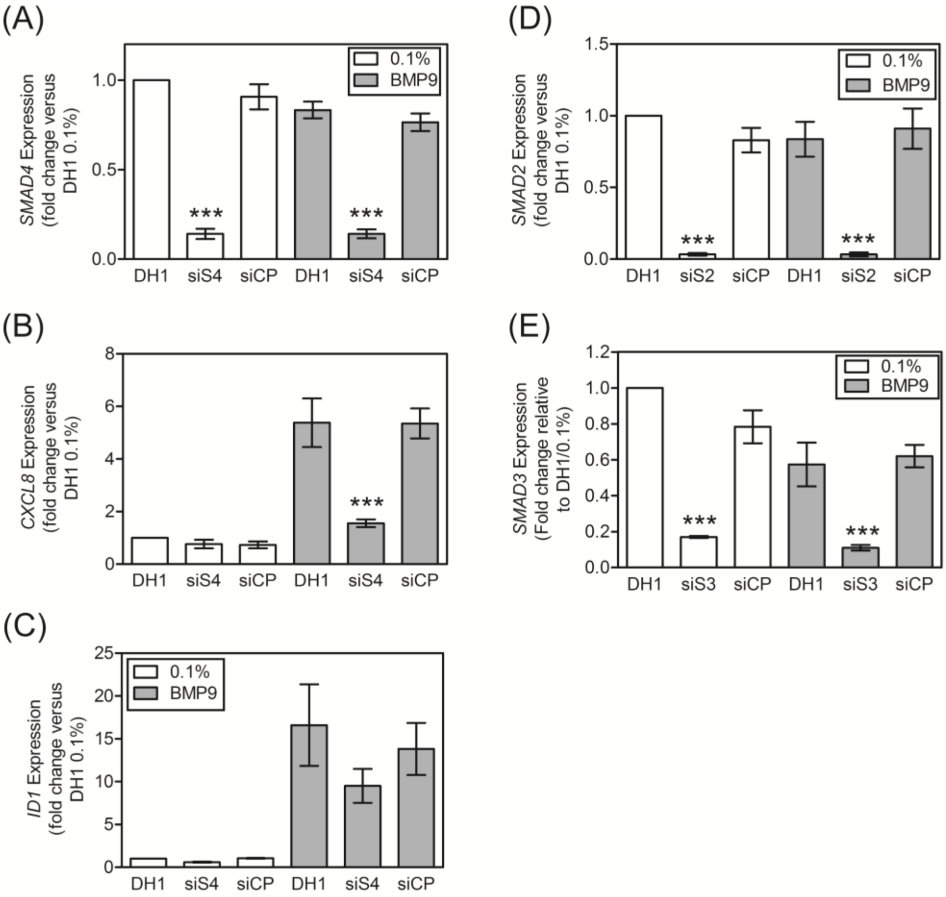

Figure S2. Confirmation of the Functional Impact of SMAD4 siRNAs and Knockdown Efficiency of SMAD2 and SMAD3 siRNAs.

HPAECs were transfected with siRNA for (A-C) *SMAD4* (siS4) (D) *SMAD2* (siS2), (E) *SMAD3* (siS3) or a non-targeting control pool (siCP) using DharmaFECT1™ (DH1). Cells were serum-restricted and then treated with 1ng/ml BMP9 in 0.1% FBS (0.1%) for 8 h for mRNA. (A-C) cDNA for siS4 transfected cells was analysed by QPCR for expression of (A) *SMAD4*, (B) *CXCL8* and (C) *ID1*. (D) cDNA from siS2 transfected cells was analysed for *SMAD2* expression. (E) cDNA from siS3 transfected cells was analysed for *SMAD3* expression. Expression data, normalised to *ACTB*, are presented as the fold change relative to DH1/0.1% and are mean ± SEM of 3 separate experiments. ****P*<0.001 compared to siCP with same treatment, students t-test.

Journal of Cell Science • Supplementary information

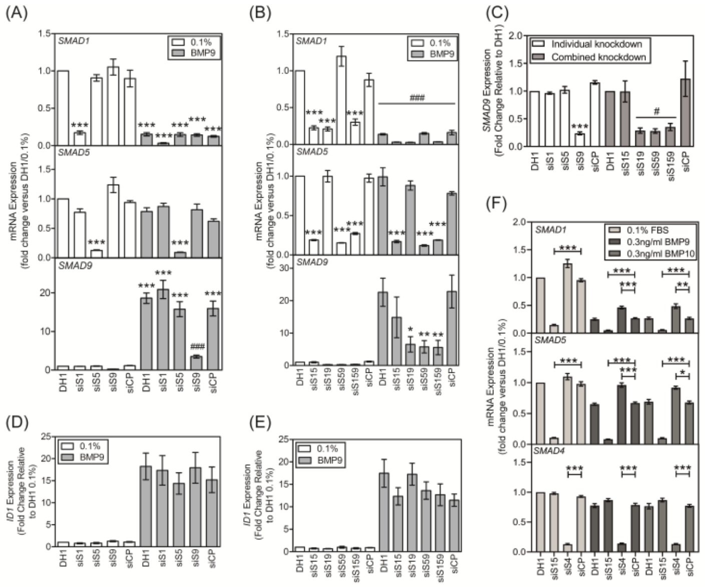

Figure S3. Confirmation of the Selectivity of siRNAs for *SMAD1*, *SMAD5*, *SMAD9* and *SMAD4*. (A-E) HPAECs were transfected with siRNAs for *SMAD1* (siS1), *SMAD5* (siS5) or *SMAD9* (siS9) (A) alone (n=5 experiments), or (B) in combination (n=4 experiments) using DharmaFECT1™ (DH1). In parallel, cells were transfected with a non-targeting control pool (siCP). Cells were then serum-restricted and treated with 1ng/ml BMP9 in 0.1% FBS (0.1%) for 8 h for mRNA. QPCR data, normalized to *ACTB*, are the fold change relative to DH1/0.1% and are mean ± SEM. (C) Baseline SMAD9 knockdown data extracted from (A) and (B). (D and E) *ID1* induction by BMP9 in HPAECs transfected with (D) individual *SMAD* siRNAs or (E) *SMAD* siRNAs in combination. *P<0.05, **P<0.01 ***P<0.001 compared to siCP/0.1% FBS, #P<0.05 and ###P<0.001 compared to siCP/BMP9, one-way repeated measures ANOVA with *post-hoc* Tukey's HSD test. (F) HPAECs were transfected with combined siRNAs for *SMAD1* and *SMAD5* (siS15) or siRNA for *SMAD4* (siS4) (n=5 experiments). Cells were then serum-restricted and treated with 0.3ng/ml BMP9 or BMP10 in 0.1% FBS (0.1%) for 8 h for mRNA. QPCR data, normalized to *ACTB*, are the fold change relative to DH1/0.1% and are mean ± SEM. *P<0.05, **P<0.01 ***P<0.001 compared to siCP/0.1% FBS, one-way repeated measures ANOVA with *post-hoc* Sudak test.

Journal of Cell Science • Supplementary information

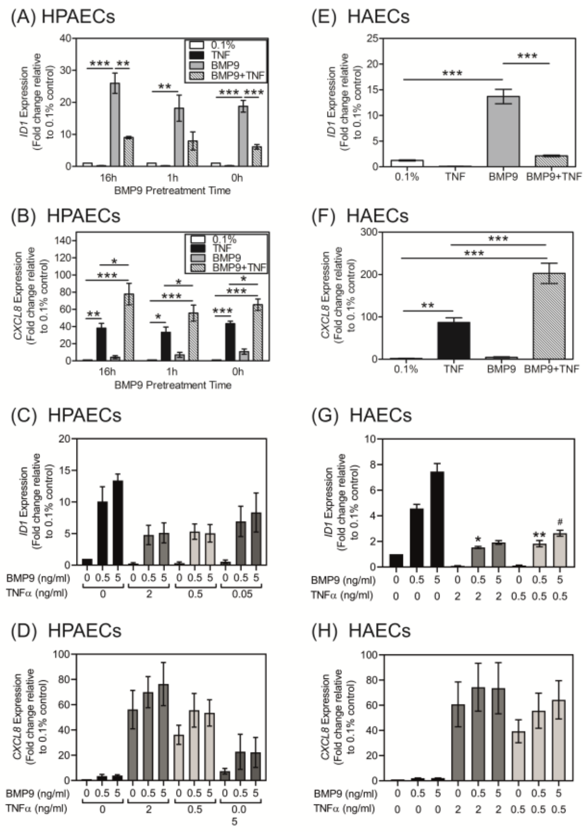

Figure S4. TNF-α Reduces BMP9-Dependent *ID1* Transcription While BMP9 enhances CXCL8 Expression in Endothelial Cells

(A-D) HPAECs or (E-H) HAECs were plated, grown to confluence and serum-restricted. (A-B) HPAECs were treated with BMP9 (5 ng/ml) alone, or with TNF-α (5 ng/ml) in 0.1% FBS for 6 h. Co-treatments were added without BMP9 preincubation, or after 1h or 16 h preincubation with 5ng/ml BMP9. The expression of (A) *ID1* or (B) *CXCL8* was determined and normalised to *ACTB*. Data are mean ± sem for n=3 experiments. **P*<0.05, ***P*<0.01, ****P*<0.001, one-way repeated measures ANOVA with *post-hoc* Tukey's HSD test. (C-D) HPAECs were treated with BMP9 (0.5, 5 ng/ml) alone, or with TNF-α (0.05, 0.5 and 2ng/ml) in 0.1% FBS for 6 h. The expression of (C) *ID1* or (D) *CXCL8* was determined and normalised to *ACTB*. Data are mean ± sem for n=3 experiments. (E-F) Confluent serum-restricted HAECs were treated with BMP9 (5 ng/ml) alone, or with TNF-α (5 ng/ml) in 0.1% FBS for 6 h. The expression of (E) *ID1* or (F) *CXCL8* was determined and normalised to *ACTB*. Data are mean ± sem for n=4 experiments. ***P*<0.01 and ****P*<0.001, one-way

Journal of Cell Science • Supplementary information

repeated measures ANOVA with *post-hoc* Tukey's HSD test. (G-H) HAECs were treated with BMP9 (0.5, 5 ng/ml) alone, or with TNF-α (0.05, 0.5 and 2ng/ml) in 0.1% FBS for 6 h. The expression of (G) *ID1* or (H) *CXCL8* was determined and normalised to *ACTB*. Data are mean ± sem for n=3 experiments. **P*<0.05 and ***P*<0.01 with respect to 0.5ng/ml BMP9, $^{\#}P<0.05$ with respect to 5ng/ml BMP9 , Friedman analysis test with post-hoc Dunn's multiple comparison test.

Journal of Cell Science • Supplementary information
