# Supplementary material for: Endothelial protective factors BMP9 and BMP10 inhibit CCL2 release by human vascular endothelial cells
Source: J Cell Sci. 2020 Jul 21;133(14):jcs239715. doi: 10.1242/jcs.239715 (PMC7390625; doi:10.1242/jcs.239715)
Supplement: Reviewer comments [file joces-133-239715_review_history.pdf]

The Endothelial Protective Factors, BMP9 and BMP10, Inhibit CCL2 Release By Human Vascular Endothelial Cells

Paul D. Upton, John E. S. Park, Patricia M. De Souza, Rachel J. Davies, Mark J. D. Griffiths, Stephen J. Wort and Nicholas W. Morrell

DOI: 10.1242/jcs.239715

Editor: John Heath

Review timeline

Original submission:	30 September 2019
Editorial decision:	31 October 2019
First revision received:	31 March 2020
Accepted:	10 June 2020

Original submission

First decision letter

MS ID#: JOCES/2019/239715

MS TITLE: The Endothelial Protective Factors, BMP9 and BMP10, Inhibit CCL2 Release By Human Vascular Endothelial Cells

AUTHORS: Paul D Upton, John E.S. Park, Patricia M De Souza, Rachel J Davies, Mark J.D. Griffiths, Stephen J Wort, and Nicholas W Morrell
ARTICLE TYPE: Research Article

We have now reached a decision on the above manuscript.

To see the reviewers' reports and a copy of this decision letter, please go to: https://submit-jcs.biologists.org and click on the 'Manuscripts with Decisions' queue in the Author Area. (Corresponding author only has access to reviews.)

As you will see, the reviewers raise a number of substantial criticisms that prevent me from accepting the paper at this stage. They suggest, however, that a revised version might prove acceptable, if you can address their concerns. If you think that you can deal satisfactorily with the criticisms on revision, I would be pleased to see a revised manuscript. We would then return it to the reviewers.

Please ensure that you clearly highlight all changes made in the revised manuscript. Please avoid using 'Tracked changes' in Word files as these are lost in PDF conversion.

I should be grateful if you would also provide a point-by-point response detailing how you have dealt with the points raised by the reviewers in the 'Response to Reviewers' box. Please attend to all of the reviewers' comments. If you do not agree with any of their criticisms or suggestions please explain clearly why this is so.

© 2020. Published by The Company of Biologists under the terms of the Creative Commons Attribution License (https://creativecommons.org/licenses/by/4.0/).

Reviewer 1

Advance summary and potential significance to field

In the present study Upton et al. investigated the suppressive effect of primarily BMP9 on CCL2/MCP1 expression and release in human pulmonary artery endothelial cells (HPAECs). They showed that this effect was dependent on Alk1 but independent of BMPRII. Analysis of the intracellular canonical Smad signalling pathway revealed that not Smad1/5 but the co-smad, Smad4, is necessary to mediate the repression of CCL2. Of interest, BMP9 does not interfere with CCL2 induction by TNFα in human aortic endothelial cells (HAECs) or HPAECs. Overall this study supports the notion that BMP9 plays an important role for endothelial homeostasis by suppressing CCL2 and along this line monocyte activation. However, BMP9 does not interfere with a strong proinflammatory signal such as TNFα to induce CCL2 expression and release.

Comments for the author

Major comments:
In general:
The findings in the current version of the manuscript are not entirely novel, which reduces the overall interest to the readers. The authors have already shown in earlier publications that BMP9 selectively increased expression of inflammatory molecules such as IL6, IL8 and E-selectin. They have already shown that BMP9 increases Id1 and Id2 via BMPRII and Smad1/5 phosphorylation 1, which is in part recapitulated in Figure 1F, 3A and 3E. However, the interesting finding is that the mechanism for CCL2 expression/release seems to be different from IL6 and IL8 as the authors have shown that this effect is independent of Smad1/5 and BMPR2 but dependent on another type II receptor and Smad4. Overall, further analysis of Smad4-dependent regulation of CCL2 and presumably other gene expression would increase the impact of the present study. Current conclusions drawn from the presented results are unprecise and therefore over interpreted:

1) In the title the authors claim that BMP9 and BMP10 inhibit CCL2 release by human vascular endothelial cells, however, BMP10 was only investigated once in Figure 1 and most of the experiments are performed in HPAECs (only in Figure 5 HAECs are used additionally).
2) Figure 5: To examine if BMP9 really does not affect TNFα induced CCL2 expression, it is necessary to investigate different concentrations of TNFα and BMP9 on endothelial cells.
3) It is not clear to me why the authors choose to especially investigated CCL2.
As already other inflammatory molecules have been investigated1, 2, I would suggest a screening approach by using Profiler arrays (for mRNA or protein expression). This would increase the impact for the readers, as groups of pro-inflammatory cytokines and/or chemokines regulated by Smad1/5 as described for IL6, IL8 and E-Selectin could be classified versus regulation via Smad4 as shown in this manuscript for CCL2.
4) Figure 2-4: For siRNA experiments the gold standard is to use at least a second siRNA sequence targeted against the gene of interest. Hence, off-target effects of siRNA can be excluded and confidence in the data increased.
5) Figure 3: What about siRNA inhibition of ActII receptors and in combination with BMPRII inhibition to supress the BMP9 effect?
6) Besides the liver, also vascular cells produce a small amount of BMP92, 3. Is BMP9 or BMP10 expressed in HPAECs or HAECs? In this case inhibition of BMP9 or BMP10 (via siRNA or blocking antibodies) could be examined regarding CCL2 expression.

Minor comments:
Introduction:
- There is a duplication of paragraphs about Smad phosphorylation: page 3, line 18-24 is the same as on page 4, line 17-24.
Results:
- Figure 1 B (8 h)and C (24 h) are reversed on page 8, line 7 compared to Figure Legend 1 p 21, line 9.
- First paragraph Page 10, line 13: dot at the end of the sentence is missing.
Figures:
- Figure 2-4: Please quantify western blots.

© 2020. Published by The Company of Biologists under the terms of the Creative Commons Attribution License (https://creativecommons.org/licenses/by/4.0/).

- Figure 2: In my opinion supplemental Figure 2 carries important information for the readers. I would suggest adding the data panel from supplemental Figure 2 to Figure 2 in the main manuscript.
- Figure 5: Subpanels B) and C) seem to be interchanged, as C) is ELISA for CCL2 in HPAECs and B) mRNA quantification for CCL2 in HAECs. In text and legends B)
is HPAECs.
Data Supplement:
In the methods section it is referred to supplemental methods (page 6, line 20).
I cannot find the section Supplemental Methods in the data Supplement? Please add.
Supplemental Figure 1:
- B) and C): statistics for DH1/siCP versus siA1 and siB2 are missing,
respectively.

1. Upton PD, Davies RJ, Trembath RC, Morrell NW. Bone morphogenetic protein
(bmp) and activin type ii receptors balance bmp9 signals mediated by activin receptor-like kinase-1 in human pulmonary artery endothelial cells. The Journal of biological chemistry. 2009;284:15794-15804.
http://dx.doi.org/10.1074/jbc.M109.002881.
2. Young K, Conley B, Romero D, Tweedie E, O'Neill C, Pinz I, Brogan L, Lindner V, Liaw L, Vary CP. Bmp9 regulates endoglin-dependent chemokine responses in endothelial cells. Blood. 2012;120:4263-4273.
http://dx.doi.org/10.1182/blood-2012-07-440784.
3. Yao Y, Jumabay M, Ly A, Radparvar M, Wang AH, Abdmaulen R, Bostrom KI.
Crossveinless 2 regulates bone morphogenetic protein 9 in human and mouse vascular endothelium. Blood. 2012;119:5037-5047.
http://dx.doi.org/10.1182/blood-2011-10-385906.

Reviewer 2

Advance summary and potential significance to field

In this article, Paul upton and Collaborators report that:
- BMP9 and BMP10 down-regulate CCL2 expression and secretion by endothelial cells
- this effect is mediated by the ALK1 recptor, not by ALK2 or ALK3
- this effect is independent of BMPRII
- this effect requires Smad 4 but is independent of Smad1, Smad5 and Smad9
- BMP9 does not alter the induction of CCL2 by TNFalpha

Comments for the author

Although intriguing (gene regulation by BMP9 independently of Smad1/5/9 is not common), these results do not bring enough novelty to be suitable for publication in JCS.
The authors should at least:
- find the precise signaling mechanism by which BMP9 and BMP10 down-regulate CCL2 expression : which RII if not BMPRII ? is CCL2 regulated through non-canonical bmp9/10 signaling pathways (Jun-kinase, TAK, ...) ?
- find a biological function that is affected by BMP9 and/or BMP10 in endothelial cells through CCL2 down-regulation. The authors speculate about modification of vascular permeability; they should bring experimental evidence that supports this hypothesis
- perform the siRNA experiments in response to BMP10 as the results may be different from those obtained in response to BMP9.

Minor comments:
- The text on page 4, lines 17-24 is redundant with that in page 3, lines 18-24.
- Figure 1: panels (B) and (C) are inverted as compared to the comments in page 8, line 7
- Figure 1: in the legend, the statistical significance is only defined for **, whereas in the Figure some bars are labeled with * and others with ***
- Figure 2: in the legend (line 14) * is attributed to two different p values

© 2020. Published by The Company of Biologists under the terms of the Creative Commons Attribution License (https://creativecommons.org/licenses/by/4.0/).

- Figure 4: Basal Smad9 expression and its repression by siS9 should be shown.

First revision

Author response to reviewers' comments

We are grateful to the reviewers for their considered reviews and have attempted to address all of the comments and questions as fully as possible in our responses below.

Reviewer 1 Advance Summary and Potential Significance to Field: In the present study Upton et al. investigated the suppressive effect of primarily BMP9 on CCL2/MCP1 expression and release in human pulmonary artery endothelial cells (HPAECs). They showed that this effect was dependent on Alk1 but independent of BMPRII. Analysis of the intracellular canonical Smad signalling pathway revealed that not Smad1/5 but the co-smad, Smad4, is necessary to mediate the repression of CCL2. Of interest, BMP9 does not interfere with CCL2 induction by TNFα in human aortic endothelial cells (HAECs) or HPAECs. Overall this study supports the notion that BMP9 plays an important role for endothelial homeostasis by suppressing CCL2 and along this line monocyte activation. However, BMP9 does not interfere with a strong proinflammatory signal such as TNFα to induce CCL2 expression and release.

Reviewer 1 Comments for the Author:

Major comments:

In general:

The findings in the current version of the manuscript are not entirely novel, which reduces the overall interest to the readers. The authors have already shown in earlier publications that BMP9 selectively increased expression of inflammatory molecules such as IL6, IL8 and E-selectin. They have already shown that BMP9 increases Id1 and Id2 via BMPRII and Smad1/5 phosphorylation 1, which is in part recapitulated in Figure 1F, 3A and 3E. However, the interesting finding is that the mechanism for CCL2 expression/release seems to be different from IL6 and IL8 as the authors have shown that this effect is independent of Smad1/5 and BMPR2 but dependent on another type II receptor and Smad4. Overall, further analysis of Smad4-dependent regulation of CCL2 and presumably other gene expression would increase the impact of the present study. Current conclusions drawn from the presented results are unprecise and therefore over interpreted:

R1C1. In the title the authors claim that BMP9 and BMP10 inhibit CCL2 release by human vascular endothelial cells, however, BMP10 was only investigated once in Figure 1 and most of the experiments are performed in HPAECs (only in Figure 5 HAECs are used additionally).

R1C1AR. We thank the reviewer for this comment and have undertaken additional experiments to support our claim. We have included data demonstrating concentration dependent inhibition of CCL2 expression by BMP10 in Figure 1D. In addition, we have generated concentration response data for both BMP9 and BMP10 in HAECs, these data being presented in Figures 1H and 1I. For the additional experiments we have undertaken in HPAECs to explore the role of ACTR-IIA (Figures 1D-F) and Smad signalling (Figure 4F and Figure S3F), we have also included BMP10 in addition to BMP9.

R1C2. Figure 5: To examine if BMP9 really does not affect TNFα induced CCL2 expression, it is necessary to investigate different concentrations of TNFα and BMP9 on endothelial cells.

R1C1AR. We thank the reviewer for this comment. We have undertaken these experiments using two concentrations of BMP9 (0.5 and 5ng/ml) in combination with either 2ng/ml or 0.5ng/ml TNF-α in HAECs, these data are now included as Figure 5F and Figures S4G-H. We then undertook a similar analysis in HPAECs, also including an additional TNF concentration of 0.05ng/ml TNF-α. These data are presented in Figure 5C and Figures S4C-D. In summary, the responses we observed

© 2020. Published by The Company of Biologists under the terms of the Creative Commons Attribution License (https://creativecommons.org/licenses/by/4.0/).

were consistent regardless of the concentration of either ligand.

R1C3. It is not clear to me why the authors choose to especially investigated CCL2. As already other inflammatory molecules have been investigated1, 2, I would suggest a screening approach by using Profiler arrays (for mRNA or protein expression). This would increase the impact for the readers, as groups of pro-inflammatory cytokines and/or chemokines regulated by Smad1/5 as described for IL6, IL8 and E-Selectin could be classified versus regulation via Smad4 as shown in this manuscript for CCL2.

1. Upton PD, Davies RJ, Trembath RC, Morrell NW. Bone morphogenetic protein (bmp) and activin type ii receptors balance bmp9 signals mediated by activin receptor-like kinase-1 in human pulmonary artery endothelial cells. The Journal of biological chemistry. 2009;284:15794-15804. http://dx.doi.org/10.1074/jbc.M109.002881.
2. Young K, Conley B, Romero D, Tweedie E, O'Neill C, Pinz I, Brogan L, Lindner V, Liaw L, Vary CP. Bmp9 regulates endoglin-dependent chemokine responses in endothelial cells. Blood. 2012;120:4263-4273. http://dx.doi.org/10.1182/blood-2012-07-440784.

R1C3AR. We chose CCL2 as we were interested in this cytokine in relation to pulmonary arterial hypertension. Although it would be interesting to address this in a larger screen, the suggested experiment would require knocking down Smad4, Smad1, Smad5 and Smad1+5 (plus controls) in a minimum of 5 biological replicates to achieve statistical power. This would also require comparing responses in cells treated in 0.1% serum alone to those treated with BMP9. The scale of this suggested experiment (minimum 40 samples) is very large and this is an expensive undertaking just to attempt to identify other candidates. We have done this recently in another context and the cost for 33 samples was over £30000 GBP. We consider this suggestion to be beyond the scope of our current manuscript.

R1C4. Figure 2-4: For siRNA experiments the gold standard is to use at least a second siRNA sequence targeted against the gene of interest. Hence, off-target effects of siRNA can be excluded and confidence in the data increased.

R1C4AR. We agree that the ideal scenario would be to have multiple siRNAs for each individual target but our experience of testing siRNAs for these targets is that in many individual siRNAs sold by companies do not produce sufficient knockdown. For example, we have tested several individual Smad4 siRNA sequences from Sigma and Qiagen and only identified a single siRNA that led to suitably high levels of knockdown at both the protein and mRNA level. The cost of undertaking this process of every siRNA we have used would be prohibitive. Many of the siRNAs we have used have been validated in our previous publications and wherever possible, we ensure we validate the knockdown at both the proteins and mRNA level and assess the impact on related pathway members

R1C5. Figure 3: What about siRNA inhibition of ActII receptors and in combination with BMPRII inhibition to supress the BMP9 effect?

R1C5AR. We thank the reviewer for this comment. We have undertaken a comparison of the impact of knockdown of either ACTR-IIA or BMPR-II, or both receptors in combination and have assessed the impact of these on CCL2 repression by either BMP9 or BMP10. These data demonstrate that, similar to our previous data relating to Smads and IDs, combined knockdown of ACTR-IIA and BMPR- II is required to remove the inhibition of CCL2. These data are presented in Figures 1D-F.

R1C6. Besides the liver, also vascular cells produce a small amount of BMP9 2, 3. Is BMP9 or BMP10 expressed in HPAECs or HAECs? In this case inhibition of BMP9 or BMP10 (via siRNA or blocking antibodies) could be examined regarding CCL2 expression.

2. Young K, Conley B, Romero D, Tweedie E, O'Neill C, Pinz I, Brogan L, Lindner V, Liaw L, Vary CP. Bmp9 regulates endoglin-dependent chemokine responses in endothelial cells. Blood. 2012;120:4263-4273. http://dx.doi.org/10.1182/blood-2012-07-440784.
3. Yao Y, Jumabay M, Ly A, Radparvar M, Wang AH, Abdmaulen R, Bostrom KI. Crossveinless 2 regulates bone morphogenetic protein 9 in human and mouse vascular endothelium. Blood. 2012;119:5037-5047. http://dx.doi.org/10.1182/blood-2011-10-385906.

© 2020. Published by The Company of Biologists under the terms of the Creative Commons Attribution License (https://creativecommons.org/licenses/by/4.0/).

R1C6AR. We thank the reviewer for this observation, although we would dispute whether either of these publications provides robust evidence for BMP9 or BMP10 expression in endothelial cells. We have attempted to detect BMP9 and BMP10 expression in vascular endothelial cells, but no signal is detected when compared to liver (BMP9 positive control) or right atrium (BMP10 positive control) ELISA measurement of endothelial conditioned media is also negative for BMP9. This contrasts with our measurements in human plasma, where we detect 150-700pg/ml in control individuals (Hodgson et al 2019 Am.J.Respir.Crit.Care Med) Given that the EC50 of BMP9 to stimulate endothelial cell signalling is 50pg/ml, these data suggests that plasma is the primary source of endothelial-cell activating BMP9.
In Young et al. the expression of BMP9 is detected in endothelial cells that the authors stably transfected with a BMP9 expression vector, whereas no BMP9 signal is present in the control cells (Figure 2A in that paper).
Although Yao et al report they can immunoprecipitate BMP9 and CV2 from conditioned media of HAECs incubated in serum free media for 24 hours (which leads to significant cell death), they do not show that HAECs express BMP9 mRNA and do not clarify whether the cells were previously incubated in serum-containing media. The BMP9 precipitated may have been residual serum-derived BMP9 from the prior growth conditions.

Minor comments:
Introduction:
R1C7. There is a duplication of paragraphs about Smad phosphorylation: page 3, line 18-24 is the same as on page 4, line 17-24.

R1C7AR. We apologise for this duplication and have removed the duplicated paragraph from Page4, lines 17-24.

Results:
R1C8. Figure 1 B (8 h)and C (24 h) are reversed on page 8, line 7 compared to Figure Legend 1 p 21, line 9.

R1C8AR. We apologise for this oversight. We have exchanged the panels for Fig 1B and 1C in the figure panel.

R1C9. First paragraph Page 10, line 13: dot at the end of the sentence is missing.

R1C9AR. We have added the full stop.

Figures:

R1C10. Figure 2-4: Please quantify western blots.

AR10: We have quantified these blots as requested and included the data in the figures.

R1C11. Figure 2: In my opinion supplemental Figure 2 carries important information for the readers. I would suggest adding the data panel from supplemental Figure 2 to Figure 2 in the main manuscript.

R1C11AR: We thank the reviewer for this suggestion and have added these panels to Figure 2 in the manuscript as suggested.

R1C12. Figure 5: Subpanels B) and C) seem to be interchanged, as C) is ELISA for CCL2 in HPAECs and B) mRNA quantification for CCL2 in HAECs. In text and legends B) is HPAECs.

R1C12AR: We apologise for this labelling error, we have relabelled panels B and C in the Figure accordingly.

Data Supplement:
R1C13. In the methods section it is referred to supplemental methods (page 6, line 20). I cannot

© 2020. Published by The Company of Biologists under the terms of the Creative Commons Attribution License (https://creativecommons.org/licenses/by/4.0/).

find the section Supplemental Methods in the data Supplement? Please add.

R1C13AR. We apologise for the omitting the upload of the Supplemental Methods during the final preparation of the previous submission. We have moved the full methods into the main manuscript. We have included a table in the data supplement listing the antibodies for Western blotting (Table S1) and have also transferred the QPCR primer details into the data supplement (Table S2).

R1C14. Supplemental Figure 1: B) and C): statistics for DH1/siCP versus siA1 and siB2 are missing, respectively.

R1C14AR: We apologise for this omission. We have included comparison to the relevant controls as requested.

Reviewer 2 Advance Summary and Potential Significance to Field: In this article, Paul upton and Collaborators report that:

- BMP9 and BMP10 down-regulate CCL2 expression and secretion by endothelial cells
- this effect is mediated by the ALK1 recptor, not by ALK2 or ALK3
- this effect is independent of BMPRII
- this effect requires Smad 4 but is independent of Smad1, Smad5 and Smad9
- BMP9 does not alter the induction of CCL2 by TNFalpha

Reviewer 2 Comments for the Author:

Although intriguing (gene regulation by BMP9 independently of Smad1/5/9 is not common), these results do not bring enough novelty to be suitable for publication in JCS.

The authors should at least:

R2C1. find the precise signaling mechanism by which BMP9 and BMP10 down-regulate CCL2 expression

R2C1AR: We thank the reviewer for this comment. We questioned whether there might be a concentration effect with regard to the response to BMP9 and the Smad-dependence. As the inhibition of CCL2 expression was evident at low concentrations (0.1ng/ml) and we had shown a dependence on ALK1, we asked whether a role for Smad1/5 might be evident in the context of a concentration of BMP9 that would only be expected to activate ALK1 (300pg/ml). In addition, we also assessed this with respect to BMP10. Intriguingly, the inhibition of CCL2 by 0.3ng/ml of BMP9 or BMP10 was reversed by cotransfection of siRNAs for Smad1 and Smad5. At this lower concentration, Smad4 siRNA still showed an effect. Therefore, we have now concluded that this inhibition is Smad1/5-dependent, but only at concentrations of BMP9 expected to stimulate ALk1. This is different to responses such as ID2, which when stimulated with 1ng/ml BMP9, are attenuated by Smad1/5 knockdown. We have incorporated these new data into (Figure 4F and Figure S3F) and have altered the discussion to reflect our new findings.

R2C2. which RII if not BMPRII ?

R2C2AR: We thank the reviewer for this comment. Based on our previous observation that ACTR-IIA and BMPR-II can compensate for the loss of each other (Upton 2009 J.Biol.Chem), we have undertaken a comparison of the impact of knockdown of either ACTR-IIA or BMPR-II, or both receptors in combination and have assessed the impact of these on CCL2 repression by either BMP9 or BMP10. These data demonstrate that, similar to our previous data relating to Smads and IDs,

© 2020. Published by The Company of Biologists under the terms of the Creative Commons Attribution License (https://creativecommons.org/licenses/by/4.0/).

combined knockdown of ACTR-IIA and BMPR-II is required to remove the inhibition of CCL2. These data are presented in Figures 1D-F.

R2C3. is CCL2 regulated through non-canonical bmp9/10 signaling pathways (Jun-kinase, TAK, ...) ?

R2C3AR. We have assessed this with two experimental repeats using SP600125 (Jnk/SAPK inhibitor), U0126 (ERK1/2 inhibitor) and SB203580 (see figure for reviewer below). These inhibitors did not reverse the repression of CCL2 by BMP9

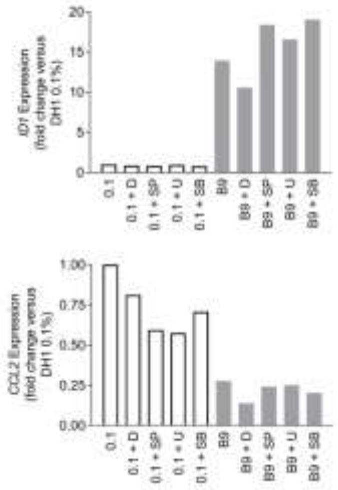

R2C4. find a biological function that is affected by BMP9 and/or BMP10 in endothelial cells through CCL2 down-regulation.

R2C4AR. We agree that a functional readout would be ideal and have collected conditioned media from endothelial cells treated with BMP9 and BMP10 to assess their effect on the migration of THP-1 cells using transwell migration assays. We had generated some initial data to show that THP-1 cell migration is reduced in conditioned media from HPAECs (n=4 biological repeats) treated with BMP9. **However, the final experiments to examine this in detail had to be stopped due to the lockdown imposed due to Covid-19. Unfortunately, the dataset below does not achieve significance though we can include this in the manuscript as a supplement figure if requested. It is currently uncertain when the additional experiments might be able to be completed.**

The Figure below shows THP1 monocytic cell transwell migration mediated by conditioned media (CM) from endothelial cells incubated with EBM2/0.1% FBS or with EBM2/0.1% FBS+1ng/ml BMP9. Briefly, conditioned media from were placed in the lower chambers of Neuroprobe chemotaxis chambers (5µm pore size, 3.2mm diameter) THP-1 cells were then seeded on top of the chamber at 50000 cells/20µl in EBM2/0.1% FBS and incubated at 37°C for 2 hours. At the end of this period, the remaining cells were aspirated and 20µL ice-cold 20mM EDTA in PBS added and the filter incubated at 4°C for 15min to release any partially migrated cells. The top of the filter was then gently flushed with PBS and then removed. To the lower chamber, 3µl of 5mg/ml MTT was added and incubated at 37°C for 90min. This was gently aspirated to avoid disturbing the cells and 20µl DMSO added to each well. The absorbance was then measured at 595nm.

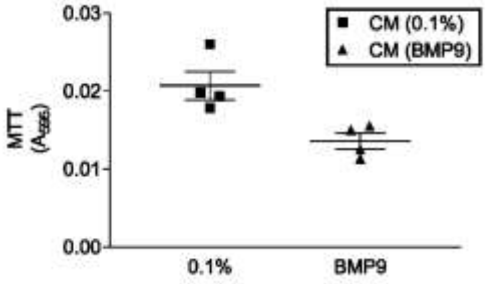

© 2020. Published by The Company of Biologists under the terms of the Creative Commons Attribution License (https://creativecommons.org/licenses/by/4.0/).

R2C5. The authors speculate about modification of vascular permeability; they should bring experimental evidence that supports this hypothesis

R2C5AR. We have been studying endothelial monolayer permeability as part of a wider assessment of acute lung injury that is currently in the review process. We have found that BMP9 elicits a very rapid signalling to reduce monolayer permeability at an early phase of treatment. As the time course of CCL2 reduction by BMP9 is at a later phase, dissecting this as a component of the regulation of permeability would be technically challenging. As detailed in R2C4AR, we have identified a direct measure of function
R5. To do

R2C6. - perform the siRNA experiments in response to BMP10 as the results may be different from those obtained in response to BMP9.

R2C6AR. As part of our further investigation of type II receptor contributions and Smad signalling, we have included BMP10, showing that in the context of CCL2, the BMP9 and BMP10 exhibit the same function. All of them? Smad4 knockdown can be done - Smad1/5/9 repeats would take a long time to do for n=5

Minor comments:

R2C7. The text on page 4, lines 17-24 is redundant with that in page 3, lines 18-24.

R2C7AR. We apologise for this duplication and have removed the duplicated paragraph from Page4, lines 17-24.

R2C8. Figure 1: panels (B) and (C) are inverted as compared to the comments in page 8, line 7

R2R8AR. We apologise for this oversight. We have changed exchanged the panels for Fig 1B and 1C in the figure panel.

R2C9. Figure 1: in the legend, the statistical significance is only defined for **, whereas in the Figure some bar are labeled with * and others with ***

R2C9AR: We apologies for this error have amended the significance parameters to refer to the correct levels of significance (*P<0.05, **P<0.01, ***P<0.001).

R2C10. Figure 2: in the legend (line 14) * is attributed to two different p values

R2C10AR. We apologise for this error and have amended the significance parameters accordingly such that *P<0.05 and **P<0.01.

R2C11. Figure 4: Basal Smad9 expression and its repression by siS9 should be shown.

R2C11AR. We have extracted the baseline expression data and presented this in Figure S3C.

<u>Second decision letter</u>

MS ID#: JOCES/2019/239715

MS TITLE: The Endothelial Protective Factors, BMP9 and BMP10, Inhibit CCL2 Release By Human Vascular Endothelial Cells

© 2020. Published by The Company of Biologists under the terms of the Creative Commons Attribution License (https://creativecommons.org/licenses/by/4.0/).

AUTHORS: Paul D Upton, John E.S. Park, Patricia M De Souza, Rachel J Davies, Mark J.D. Griffiths, Stephen J Wort, and Nicholas W Morrell
ARTICLE TYPE: Research Article

I am happy to tell you that your manuscript has been accepted for publication in Journal of Cell Science, pending standard ethics checks.

Reviewer 1

Advance summary and potential significance to field

In the present study Upton et al. investigated the suppressive effect of primarily BMP9 on CCL2/MCP1 expression and release in human pulmonary artery endothelial cells (HPAECs). They showed that this effect was dependent on Alk1 but independent of BMPRII. Analysis of the intracellular canonical Smad signalling pathway revealed that not Smad1/5 but the co-smad, Smad4, is necessary to mediate the repression of CCL2. Of interest, BMP9 does not interfere with CCL2 induction by TNFα in human aortic endothelial cells (HAECs) or HPAECs. Overall this study supports the notion that BMP9 plays an important role for endothelial homeostasis by suppressing CCL2 and along this line monocyte activation. However, BMP9 does not interfere with a strong proinflammatory signal such as TNFα to induce CCL2 expression and release.

Comments for the author

In the revised manuscript, the authors followed the suggestions of the reviewers and collected several new experimental data sets. Thus, the manuscript was substantially improved. The discussions and reasons on other suggestions that could not be implemented were understandable for me. Therefore, the manuscript is now suitable for publication in J Cell Science.

© 2020. Published by The Company of Biologists under the terms of the Creative Commons Attribution License (https://creativecommons.org/licenses/by/4.0/).
